# eGreenhouse: Robotically positioned, low-cost, open-source CO_2_ analyzer and sensor device for greenhouse applications

**DOI:** 10.1016/j.ohx.2021.e00193

**Published:** 2021-03-26

**Authors:** Elad Levintal, Kenneth Lee Kang, Lars Larson, Eli Winkelman, Lloyd Nackley, Noam Weisbrod, John S. Selker, Chester J. Udell

**Affiliations:** a Environmental Hydrology and Microbiology, The Zuckerberg Institute for Water Research, The Jacob Blaustein Institutes for Desert Research, Ben-Gurion University of the Negev, Israel; b Openly Published Environmental Sensing (OPEnS) Lab, Oregon State University, OR, United States; c Department of Electrical Engineering and Computer Science, Oregon State University, OR, United States; d Department of Horticulture, North Willamette Research and Extension Center, Oregon State University, OR, United States; e Department of Biological & Ecological Engineering, Oregon State University, OR, United States

**Keywords:** CO_2_ sensing, Agriculture, Open source hardware, Environmental sensing

## Abstract

Advances in gas sensors and open-source hardware are enabling new options for low-cost and light-weight gas sampling devices that are also robust and easy to use and construct. Although the number of studies investigating these sensors has been increasing in the last few years, they are still scarce with respect to agricultural applications. Here, we present a complete system for high-accuracy measurements of temperature, relative humidity, luminosity, and CO_2_ concentrations. The sensors suite is integrated on the previously developed HyperRail device (Lopez Alcala et al., 2019) – a reliable, accurate, and affordable linear motion control system. All measurements are logged with a location and time-stamp. The system was assembled from only off-the-shelf or 3D printable products. We deployed the system in an agricultural greenhouse to demonstrate the system capabilities.

## Highlights

• High-accuracy measurements of temperature, relative humidity, luminosity, and CO_2._
• All sensors are accurate, off-the-self, low-cost, light-weight, and open source.
• The system can be used for stationary or dynamic measurements.

## Graphical abstract

**Figure:**
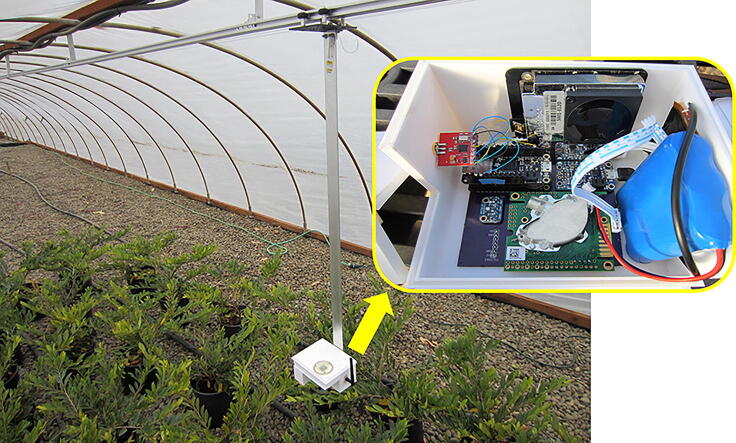

**Specifications table**

**Table:** 

Hardware name	*eGreenhouse*
Subject area	Environmental, Planetary and Agricultural Sciences
Hardware type	● Field measurements and sensors ● Electrical engineering and computer science
Open Source License	*GPL General Public License v3.0* CERN Open Hardware License
Cost of Hardware	eGreenhouse Sensor Package: $277.49 Hub: $108.39
Source File Repository	GitHub: https://github.com/OPEnSLab-OSU/eGreenHouse/tree/1D Zenodo: https://doi.org/10.5281/zenodo.4113959

## 1 Hardware in context

The spatial and temporal distribution of CO_2_ at the earth-atmosphere interface and at the boundary layer above it is of great importance for soil, agricultural and atmospheric sciences. In general, the source of elevated CO_2_ concentrations at this boundary layer depends mostly upon root respiration and soil microbial activity [2], [3], [4] and on atmospheric transport (diffusion, dispersion, and advection) [5], [6], [7], [8].

Improvement of CO_2_ gas concentration detection could lead to better understanding of agricultural productivity and the transport mechanisms at this critical interface. Accurate monitoring of CO_2_ can improve modeling and decision-making to enhance agricultural productivity [9]. Until recently, detecting CO_2_ only employed stationary sensors providing low spatial resolution [10].

The growing use of open-source hardware for research purposes creates new opportunities to bridge the gap between CO_2_ detection at high resolution and affordability. In agriculture, the use of open-source hardware and sensors for CO_2_ gas sensing is scarce. However, recent research has revealed the possible applications of operating such gas sensing systems for agricultural purposes. For example, the use of an open-source gas sensing device to detect CO_2_ concentrations in a rectangular greenhouse (106 m × 47 m) was proven by Roldán et al. [11]. Using a low-cost electrochemical CO_2_ sensor, which is a relatively low-accuracy analog sensor, they provided a CO_2_ concentration map inside a greenhouse. Their device also had temperature, relative humidity and luminosity sensors. Data from these sensors can be used for better greenhouse operation decision-making.

The aim of this work was to develop the eGreenhouse, a suite of sensors to evaluate CO_2_ concentrations and dynamics using an advanced non-dispersive infrared (NDIR) CO_2_ sensor inside a greenhouse. NDIR sensors are based on absorption spectroscopy and consist of a light source, a sample cavity and a detector [12]. We integrated this gas sensor together with temperature, relative humidity, and luminosity sensors, on a single logging device to gain high spatial and temporal resolution of the greenhouse environment. In addition, the device is small and transportable to allow deployment on a drone or a rail system to get location-tagged data throughout the greenhouse. The latter option was used to validate system performance.

## 2 Hardware description

The sensors used in this work are detailed in the next sections. First, the sections describe the sensors, then their integration on a single PCB, and finally operational instructions (software and data access). All sensors were chosen to: (1) provide accuracy similar to that of a laboratory sensor; (2) employ only off-the-shelf, open source sensors; (3) be low-cost compared to existing sensors; and (4) result in a lightweight system. All enclosures and fixtures were designed as 3D printable to allow broadly accessible replication.

A linear-motion actuator (HyperRail) positioned the sensor system [1]. The eGreenhouse includes two main components, the sensor package which is mounted on the HyperRail and the static hub from which the user can control the HyperRail and upload the data online (e.g., GoogleSheets)

The eGreenhouse can also be used in the following settings:

● Mounted on a drone for large area measurements (not necessarily within greenhouses).
● Static measurements within caves, boreholes or underground cavities in which CO_2_ concentration regimes are of interest, e.g. [13], [14], [15], [16].
● Adjacent to an open-source wind anemometer for a simple but efficient meteorological station with CO_2_ readings

We note that the validation data presented in section 7 was obtained using an earlier model due to specific site requirements at which the validation was done (see more information in section 7). Below we present the hardware design that is similarly configured (e.g., K30 for CO_2_ and SHT31-D for temperature and relative humidity), but with some minor improvements (re-designed data logger board).

## 3 Design files

**Design Files Summary**

**Table:** 

Design file name	File type	Open source license	Location of the file
*eGH*	BRD	*CERN Open Hardware License*	https://doi.org/10.5281/zenodo.4113959
*eGH*	SCH	*CERN Open Hardware License*	https://doi.org/10.5281/zenodo.4113959
*Code*	C++	*GNU General Public License v3.0*	https://doi.org/10.5281/zenodo.4113959

**eGH.brd:** BRD file that can be loaded into AutoDesk EAGLE to get the layout of the eGreenhouse PCB.

**eGH.sch**: SCH file that can be loaded into AutoDesk ENGLE to get the schematic of the circuit for the eGreenhouse PCB.

## 4 Bill of materials

### 4.1 Materials for eGH_Sensor_Package

**Table:** 

Designator	Component	Number	Cost per unit $	Total cost$	Source of materials	Material type
DevelopmentBoard	Adafruit Feather M0 with RFM95 LoRa Radio − 900 MHz - RadioFruit	1	$34.95	$34.95	Adafruit	Non-specific
Antenna Kit	900Mhz Antenna Kit - For LoPy, LoRa, etc	1	$12.75	$12.75	Adafruit	Non-specific
Antenna Connector	uFL SMT Antenna Connector	1	$0.75	$0.75	Adafruit	Non-specific
Data Loggerwith RTC Board	Adalogger FeatherWing - RTC + SD Add-on For All Feather Boards	1	$8.95****	$8.95****	Adafruit	Non-specific
OPEnS Data Logger with RTC Board	Hypnos	1	$33.04****	$33.04****	OPEnS Lab	Non-specific
Micro SD Card	16 GB MicroSD Card	1	$6.19	$6.19	Amazon	Non-specific
CO_2_ Sensor	K30 10,000 ppm CO_2_ Sensor	1	$85.00 ***	$85.00	CO2Meter	Non-specific
Lux Sensor	Adafruit TSL2591 High Dynamic Range Digital Light Sensor	1	$6.95	$6.95	Adafruit	Non-specific
Temperatureand HumiditySensor	Adafruit Sensirion SHT31-D - Temperature & Humidity Sensor	1	$13.95	$13.95	Adafruit	Non-specific
Power Convertor	PowerBoost 1000 Charger - Rechargeable 5 V Lipo USB Boost @ 1A − 1000C	1	$19.95	$19.95	Adafruit	Non-specific
Sensor PCB	Custom Component	1	$1.00*	$23.00*** including shipping & minimum order	PCBWay	FR-4
Short Male Headers	Short Feather Male Headers − 12-pin and 16-pin Male Header Set	2	$0.50	$1.00	Adafruit	Non-specific
Short Female Headers	Short Headers Kit for Feather − 12-pin + 16-pin Female Headers	1	$1.50	$1.50	Adafruit	Non-specific
Battery	Lithium Ion Battery Pack − 3.7 V 6600mAh	1	$29.50	$29.50	Adafruit	Ion

Pricing Notes

* price will vary with source

** optional

*** price will vary with capacity

**** must have either one of them

Additional equipment that may be needed:

Soldering station for soldering sensor and other parts to PCB

### 4.2 Materials for Hub

**Table:** 

Designator	Component	Number	Cost per unit $	Total cost$	Source of materials	Material type
DevelopmentBoard	Adafruit Feather M0 with RFM95 LoRa Radio − 900 MHz - RadioFruit	1	$34.95	$34.95	Adafruit	Non-specific
Antenna Kit	900Mhz Antenna Kit - For LoPy, LoRa, etc	1	$12.75	$12.75	Adafruit	Non-specific
Antenna Connector	uFL SMT Antenna Connector	1	$0.75	$0.75	Adafruit	Non-specific
Ethernet Connector	Adafruit Ethernet FeatherWing	1	$19.95	$19.95	Adafruit	Non-specific
Battery	Lithium Ion Battery Pack − 3.7 V 6600mAh	1	$29.50	$29.50	Adafruit	Ion
Short Female Headers	Short Headers Kit for Feather − 12-pin + 16-pin Female Headers	1	$1.50	$1.50	Adafruit	Non-specific
Ethernet Cable	6ft Ethernet Cable	1	$8.99	$8.99*	Amazon	Non-specific

Pricing Notes

* optional

Additional equipment that may be needed:

Soldering station for soldering development board and Ethernet Connector

## 5 Build instructions

Here we provide building instructions for each individual part and then demonstrate prototype wiring using the Sensor PCB. Additional information, instructions and updates, such as the PCB design and connections can be found at https://github.com/OPEnSLab-OSU/eGreenHouse/wiki (eGreenhouse tab)

### 5.1 eGreenhouse sensor package individual hardware components setup

All steps in this section require a soldering iron and solder. Follow typical safety protocols for soldering to prevent burns and inhalation of lead solder or flux/rosin solder: eye protection, heat resistant work surface, clamps to hold materials to be soldered, ventilation, etc.

#### 5.1.1 Development board

**Fig. 1:**
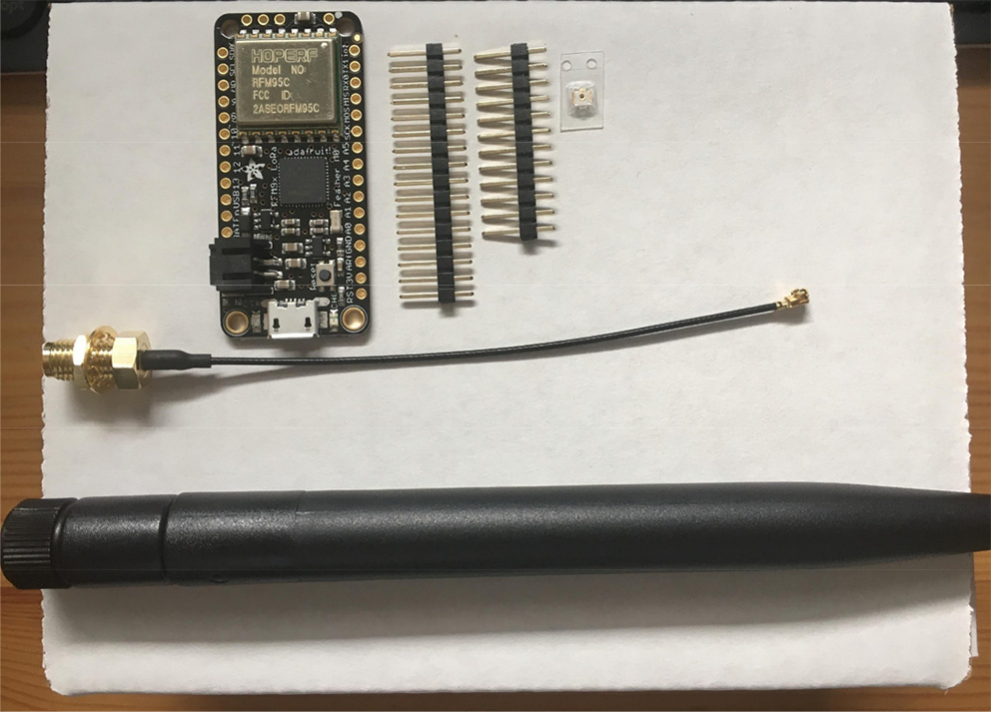
The materials that are required for the Development Board setup.

In this step, you need the Antenna Connector, Antenna Kit, development board, and 12-pin + 16-pin male headers that came with the development board (Fig. 1).

**Fig. 2:**
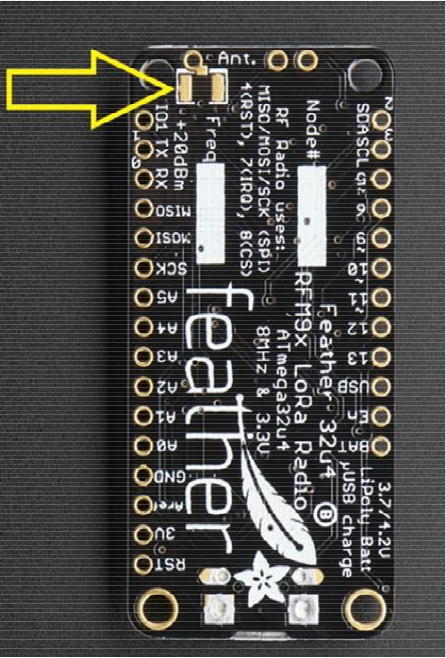
Bottom side of the Development Board as received from vendor. The Yellow arrow indicates where to solder the Antenna Connector.

**Fig. 3:**
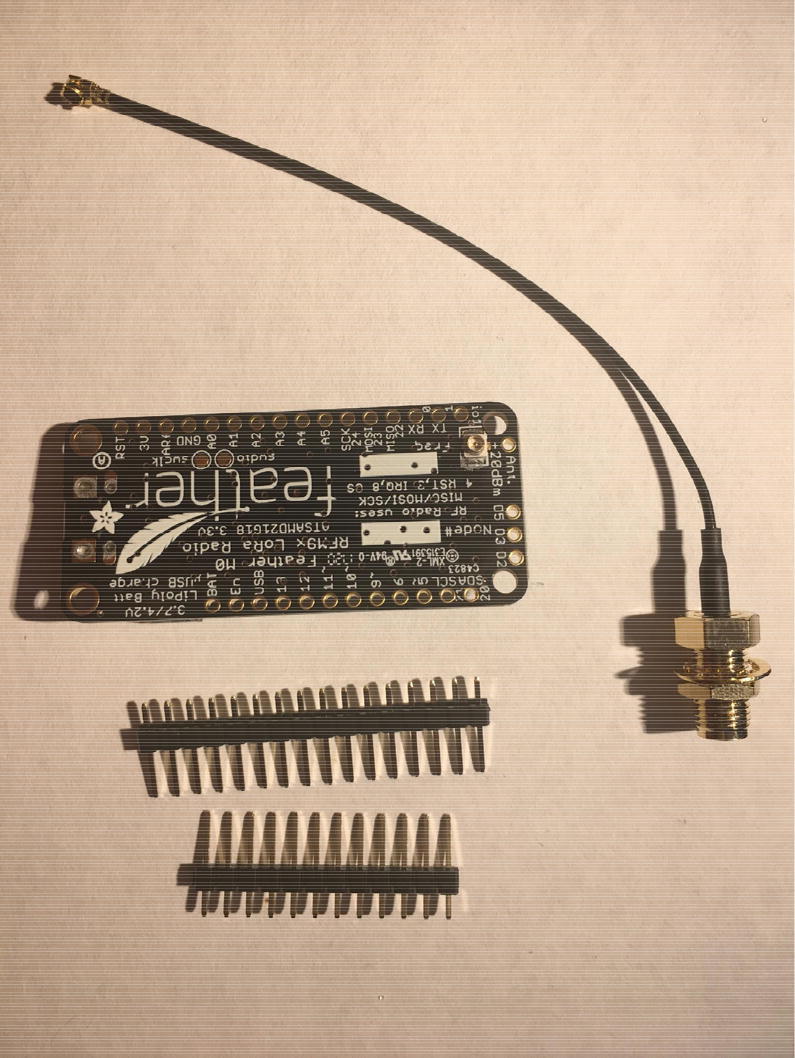
Development board with accessories after soldering the Antenna Connector on the board (see the yellow arrow at Fig. 2 for the soldering location).

First, solder the Antenna Connector in the designated location on the board. Fig. 2 with a yellow arrow will tell where to solder the Antenna Connector and Fig. 3 shows the soldered Antenna Connector.

**Fig. 4:**
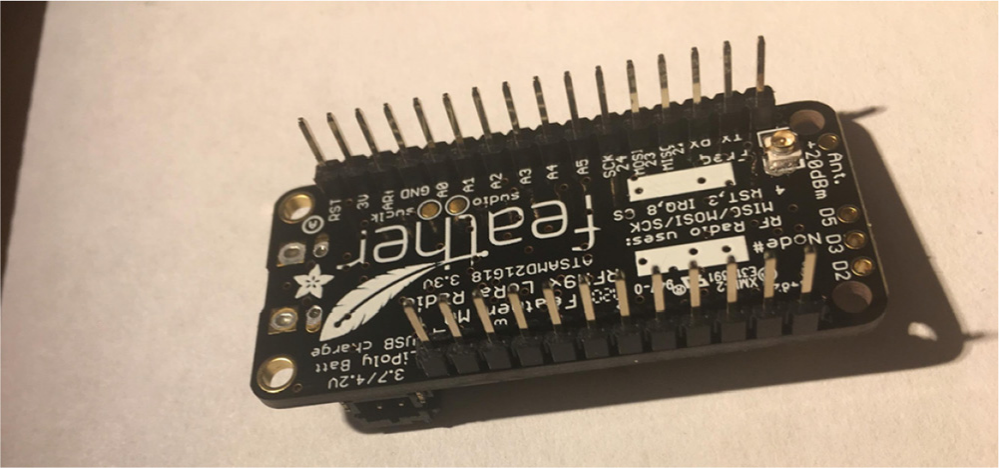
Bottom of the Development board with both male headers soldered on.

Once that step is complete, solder 12-pin + 16-pin male headers (Fig. 4). Make sure that the long side of the male headers are facing down while the short side of the male headers go through the board towards the top.

**Fig. 5:**
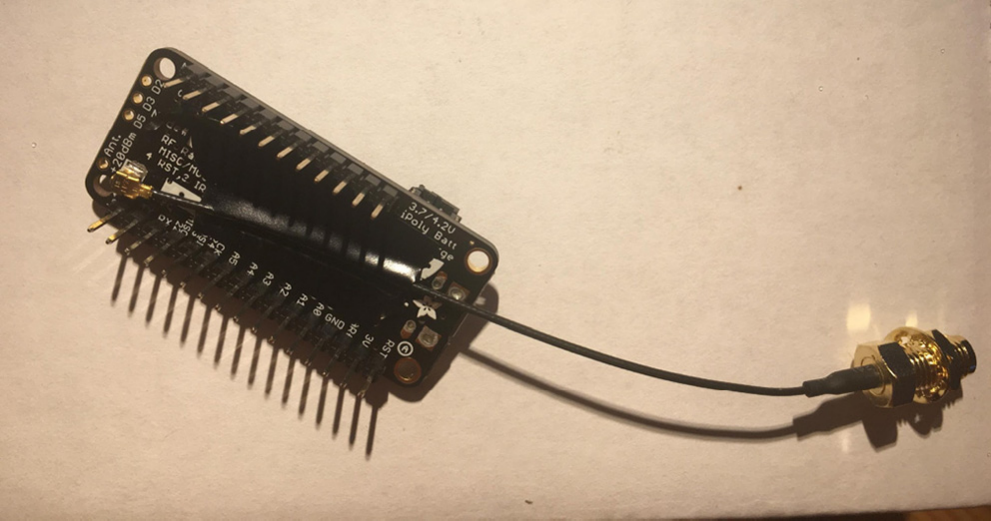
The finished development board for the eGreenhouse sensor package.

Connect the Antenna Kit, except the actual plastic antenna, on the Antenna Connector. Because it is easy for the Antenna Kit to be disconnected from the Antenna Connector, secure (strain relief) the wire for the Antenna Kit to the Development Board with either electrical tape or hot glue so that it will stay in position (Fig. 5).

#### 5.1.2 Data logger with RTC board

As shown from the bill of materials, there is an option to use either a RTC Adalogger from Adafruit or the OPEnS Data Logger with RTC Board that was created from OPEnS Lab. We will be using the Hypnos board rather than Adalogger. If you are going to use the Hypnos board, then reference here.

**Fig. 6:**
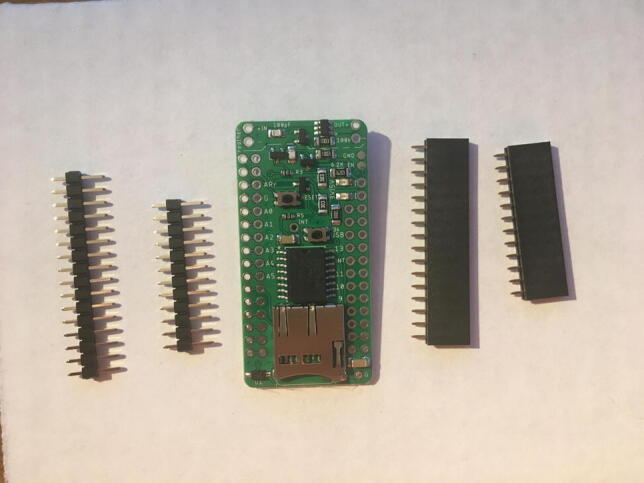
The materials for setting up the Data Logger with the RTC Board (cell battery and Micro SD card not shown).

For this step, you will need the Hypnos board, 12-pin + 16-pin male headers, 12-pin + 16-pin female headers, coin cell battery, and Micro SD card (Fig. 6). If you are planning to use Adalogger Adafruit as Data Logger with RTC Board, then you need Adalogger board, 12-pin + 16-pin stacking headers, coin cell battery, and Micro SD Card.

**Fig. 7:**
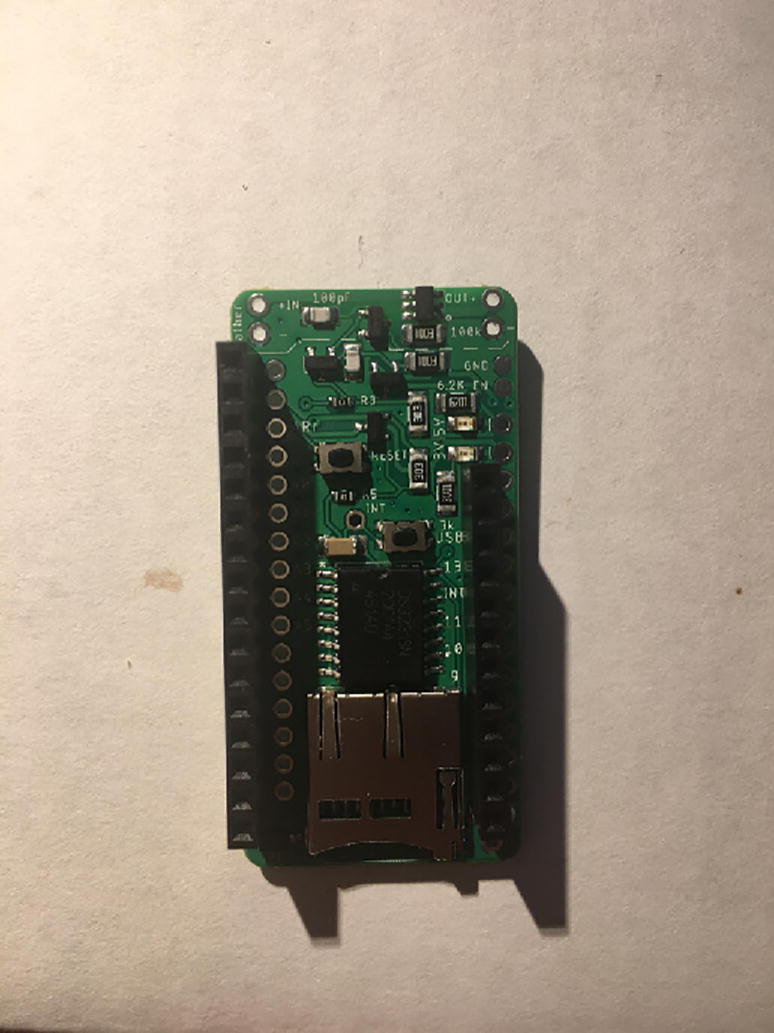
Top view of the finished Data Logger with RTC Board.

Solder the female headers on the top of the board (side with microSD slot) where it has a label of “feather” (Fig. 7).

**Fig. 8:**
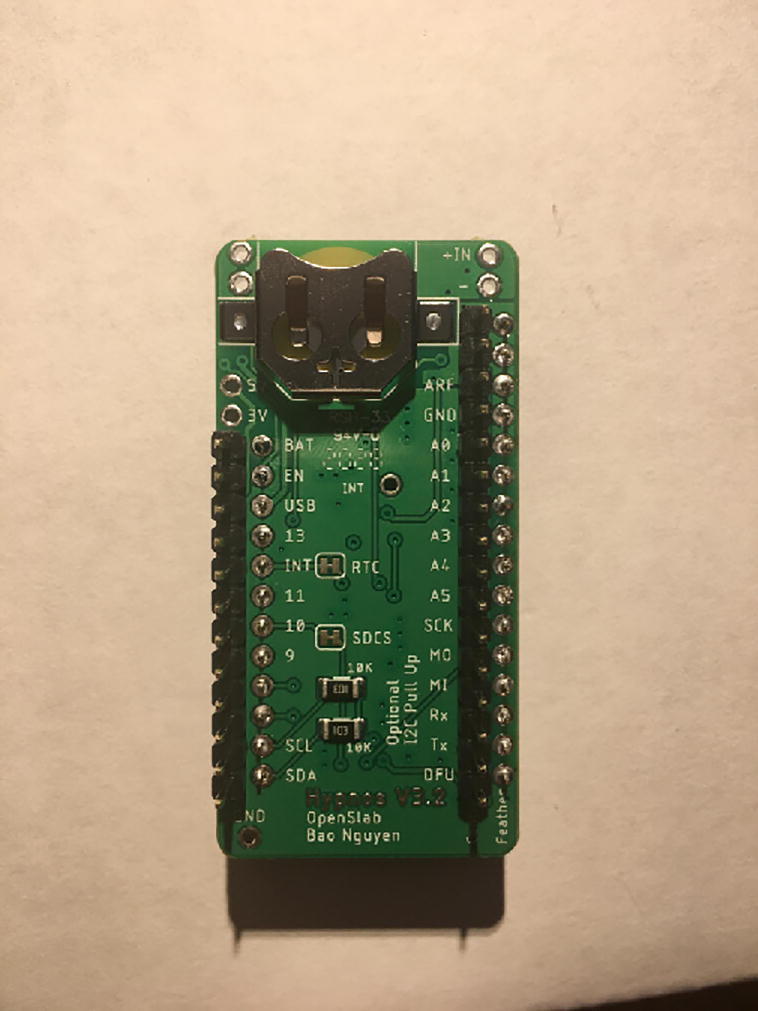
The top view of the male headers soldered into the bottom of the Data Logger with the RTC Board.

**Fig. 9:**
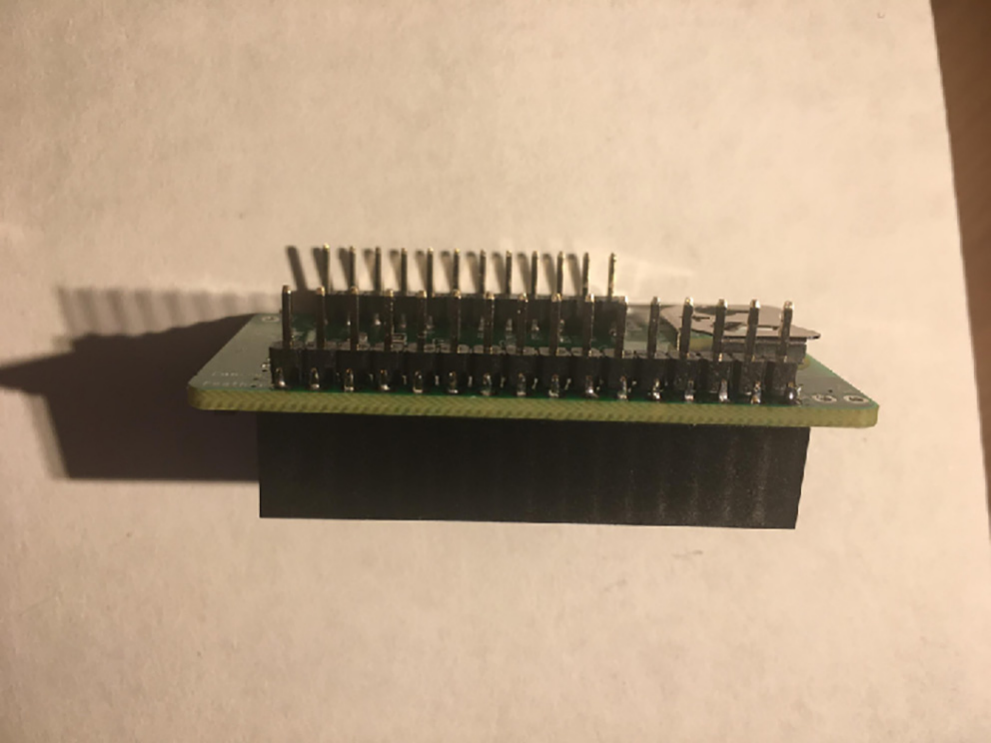
Side view of the Data Logger with the RTC Board with both male and female headers soldered on. Insert the Micro SD card into the Micro SD card holder and the coin cell battery.

Once female headers are soldered, then solder the male headers such that the long male headers are facing out on the bottom (side with coin cell battery holder) (Fig. 8, Fig. 9).

#### 5.1.3 CO_2_ sensor

**Fig. 10:**
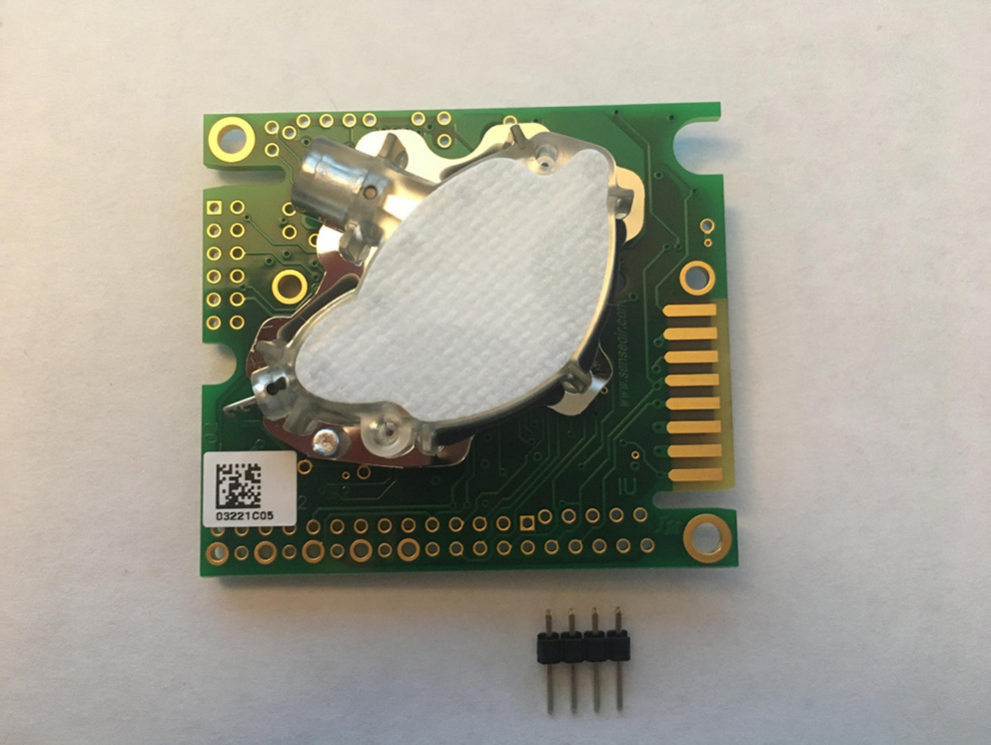
The CO_2_ Sensor and 4-pin male headers.

For this step, you need the K30 CO_2_ Sensor and 4-pin male headers (Fig. 10). The 4-pin male headers can be created by breaking the 12-pin male header into a 4-pin male header.

**Fig. 11:**
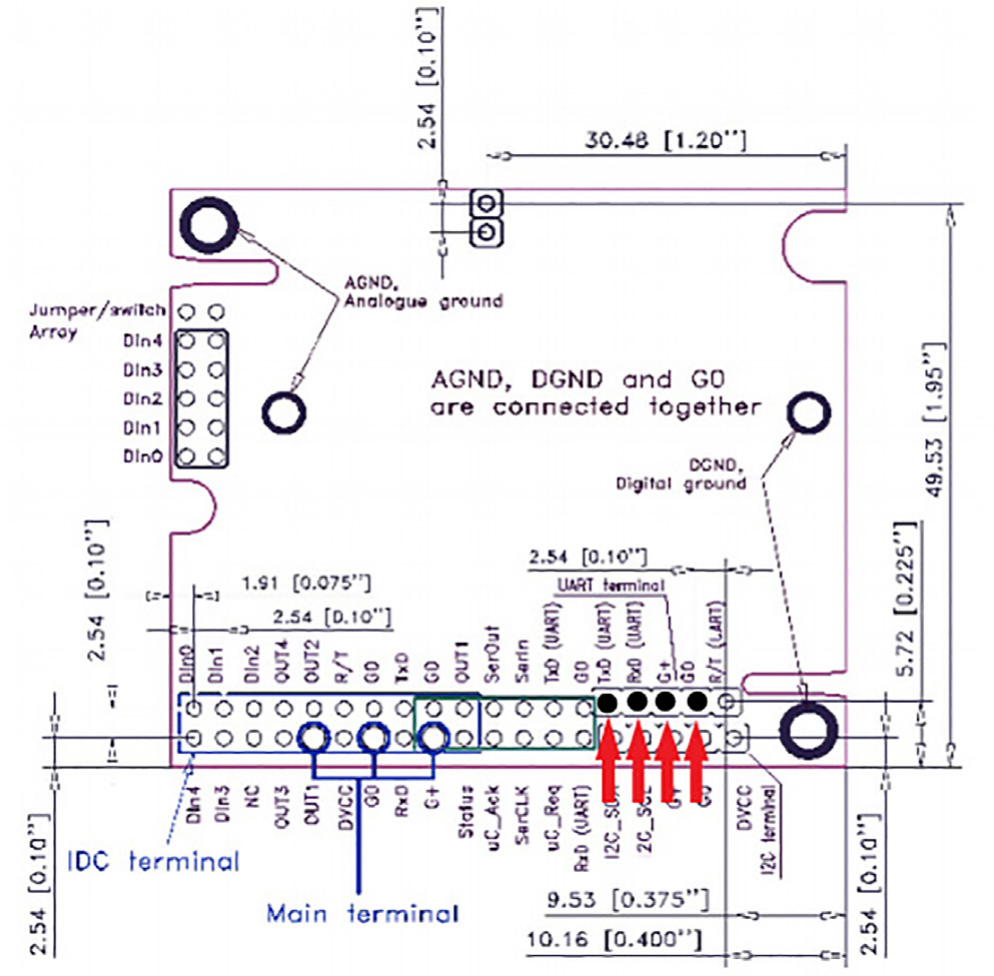
The CO_2_ Sensor layout with red arrows indicating where to solder the 4-pin male headers.

**Fig. 12:**
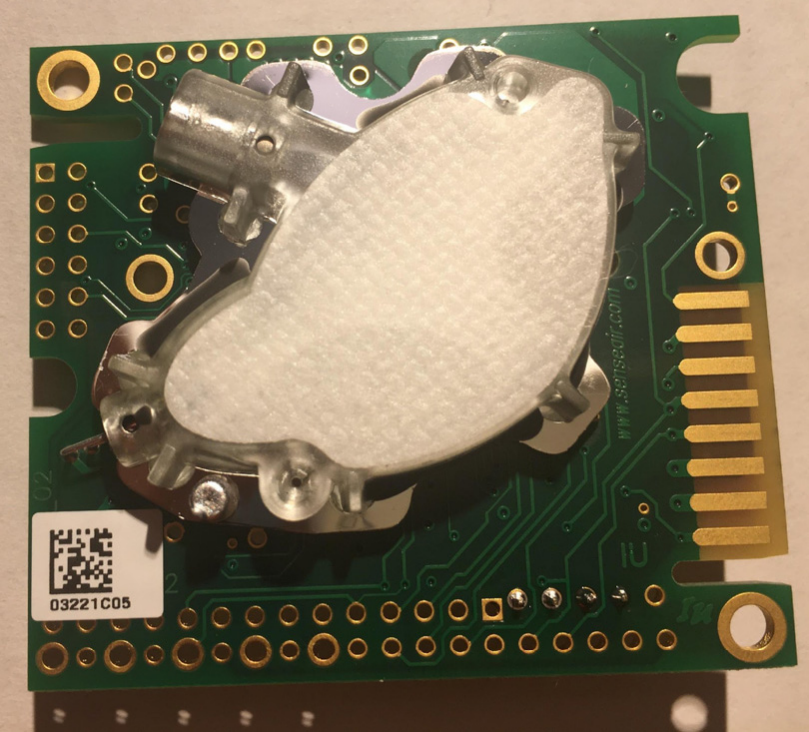
Top view of the CO_2_ Sensor after soldering 4-pin male headers.

**Fig. 13:**
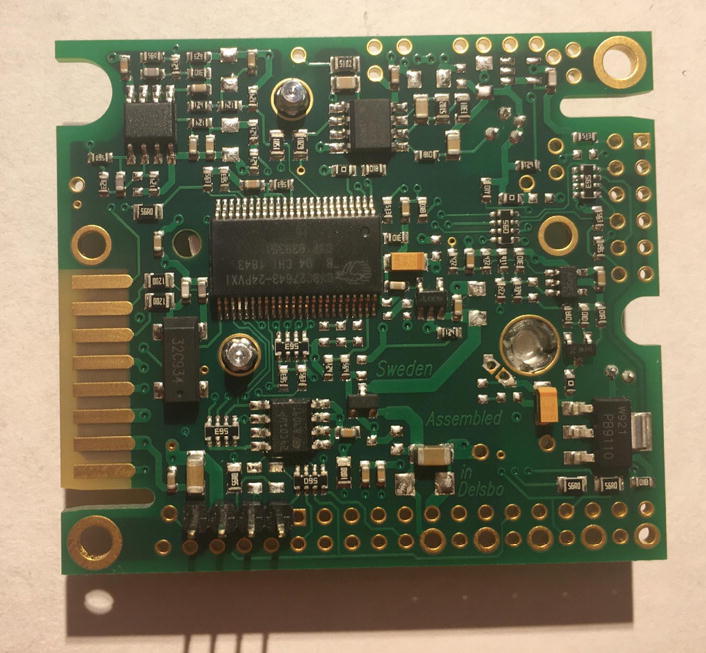
Bottom view of the CO_2_ sensor after soldering the 4-pin male headers.

Solder the 4-pin male headers on the four black dots shown by the red arrows in Fig. 11. The soldered CO_2_ Sensor is shown in Fig. 12, Fig. 13.

#### 5.1.4 PowerBoost hardware

**Fig. 14:**
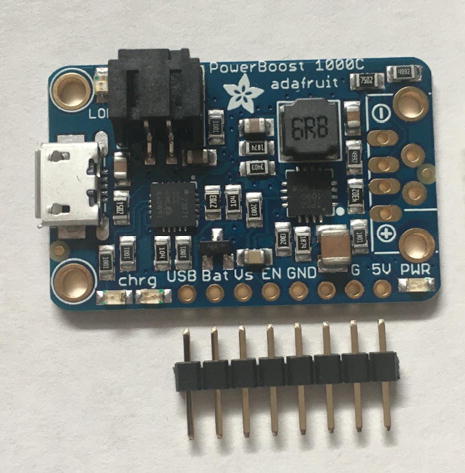
The PowerBoost.

For this step, you need the PowerBoost without the USB Type-A adapter and 8-pin male headers (Fig. 14). The USB Type-A adapter will not be used in this project. The 8-pin male headers are the remaining male-pins after using the 4-pin male headers from the CO_2_ Sensor.

**Fig. 15:**
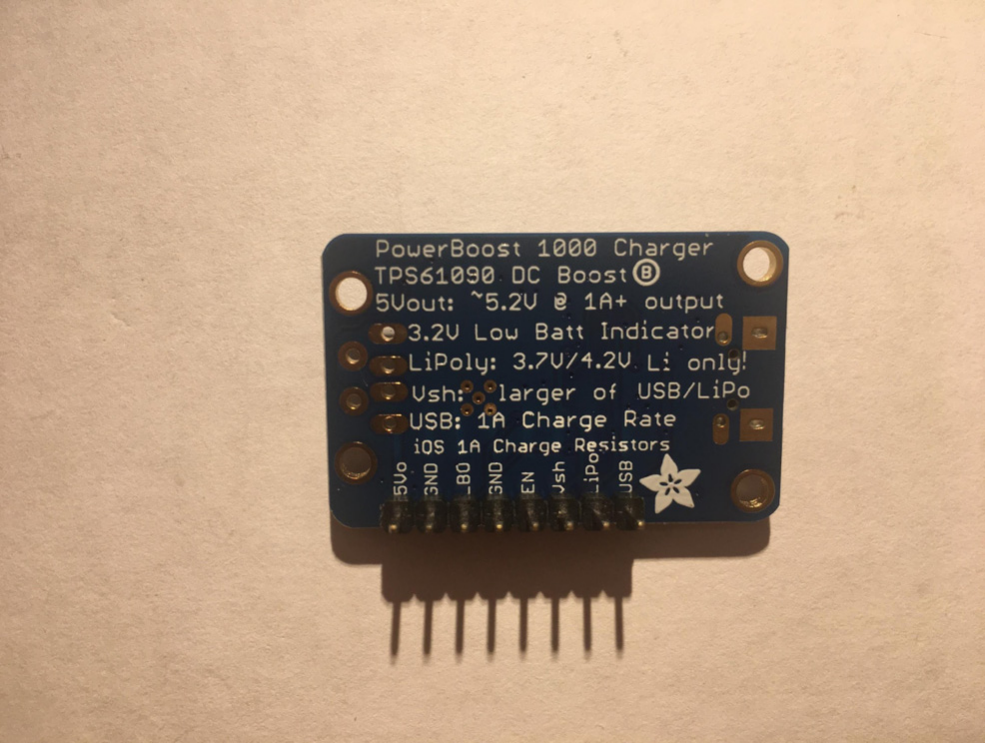
The bottom view after soldering the 8-pin male headers on the PowerBoost.

Solder the 8-pin male headers such that the long pin will be projecting out of the bottom of the board (Fig. 15).

#### 5.1.5 Lux sensor

**Fig. 16:**
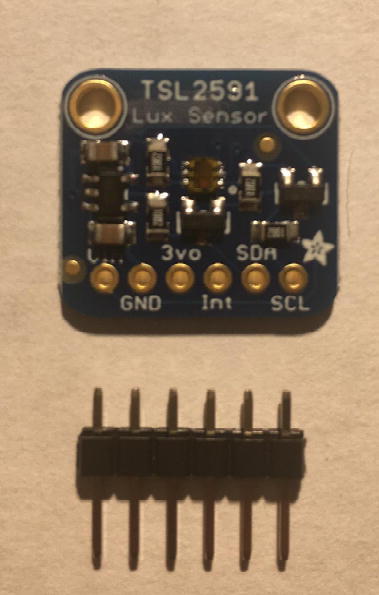
Top side of Lux Sensor and 6-pin male headers.

For this step, you need Lux Sensor and 6-pin male headers (Fig. 16). You can create the 6-pin male headers from the 16-pin male headers by breaking it to 6-pin male headers.

**Fig. 17:**
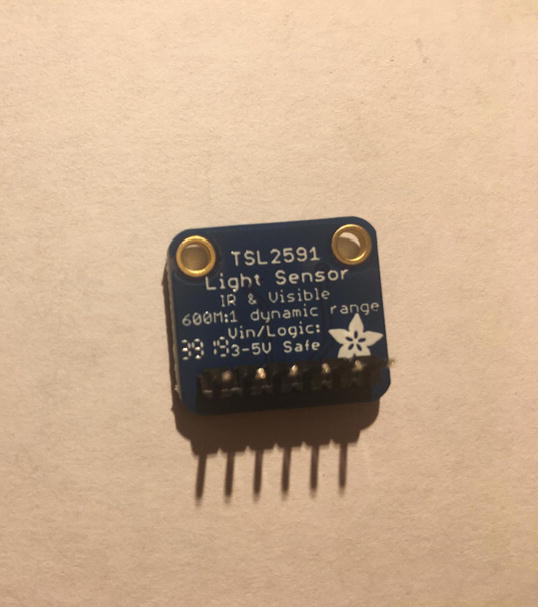
Finished bottom side of the Lux Sensor with 6-pin male headers soldered on.

Solder the 6-pin male headers onto the PowerBoost such that the long side of the headers are on the bottom of the board (Fig. 17).

#### 5.1.6 Temperature and relative humidity sensor

**Fig. 18:**
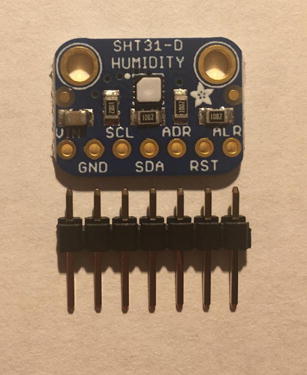
Temperature and Relative Humidity Sensor and 7-pin male headers.

For this step, you need the Temperature and Relative Humidity Sensor and 7-pin male headers (Fig. 18). You can create the 7-pin male headers from the 16-pin male headers that was used for setting up the Lux Sensor by breaking it to 7-pin male headers.

**Fig. 19:**
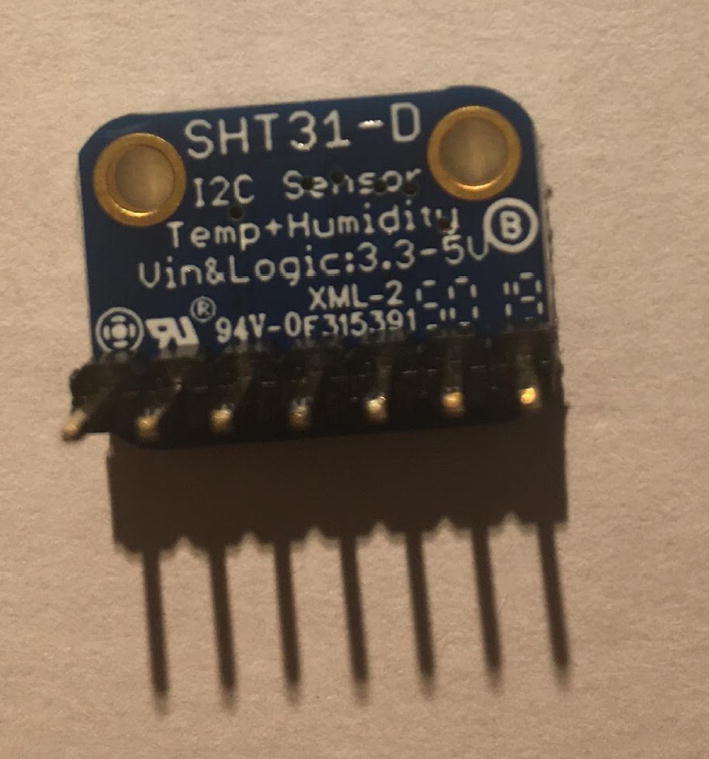
Bottom view of the finished Temperature and Relative Humidity Sensor.

Solder the 7-pin male headers onto the Temperature and Relative Humidity Sensor such that the long side of the headers are on the bottom of the board (Fig. 19).

Once this step is complete, then you are done with eGreenhouse sensor package individual hardware setup step. The next step is the assembly of the sub-systems.

### 5.2 eGreenhouse Sensor package connection setup

Steps in this section require a soldering iron and solder.

#### 5.2.1 Connect Development Board to the Data Logger with RTC Board

**Fig. 20:**
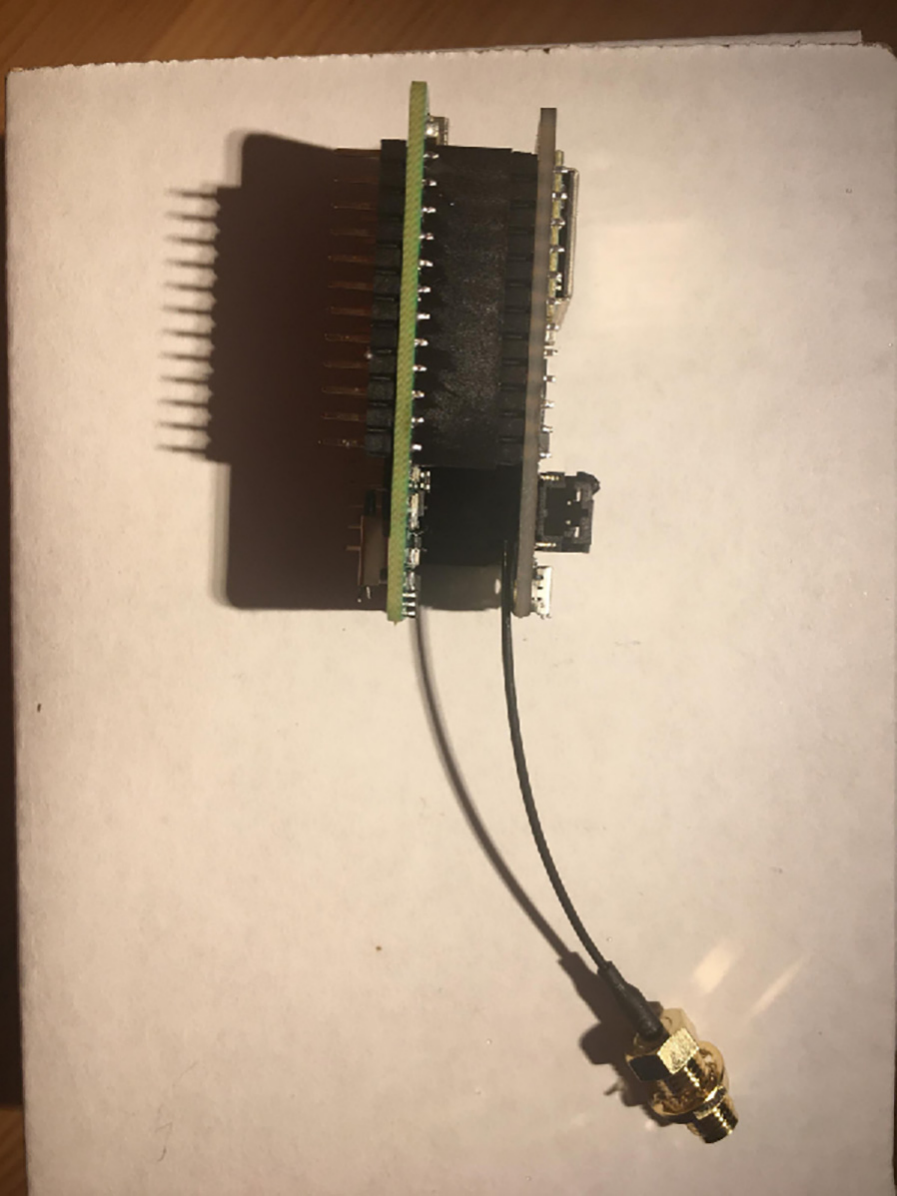
Connected Development Board and Data Logger with RTC Board on the one side.

**Fig. 21:**
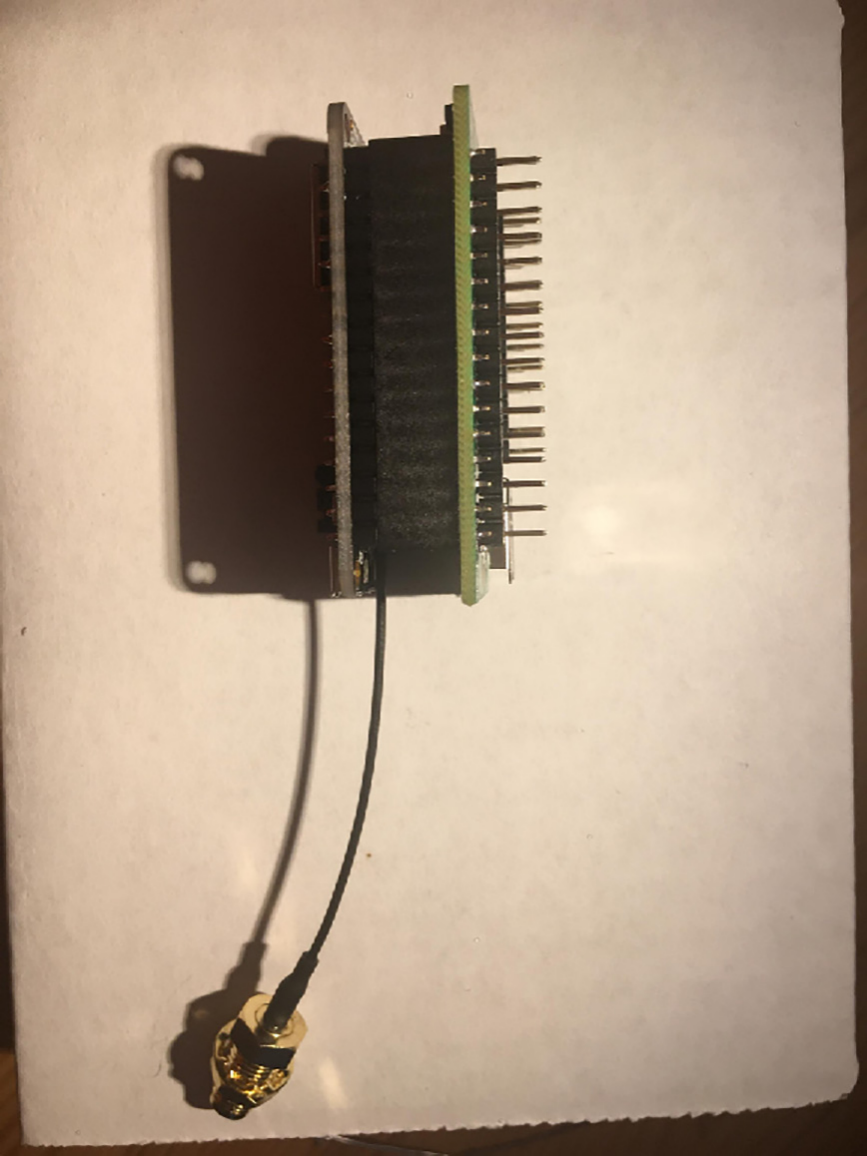
Connected Development Board and Data Logger with RTC Board on the other side.

For this step, you need the Development Board and the Data Logger with RTC Board. Connect the Development Board on top of the Data Logger with RTC Board using the headers (Fig. 20, Fig. 21).

Once they are connected, then move to the next step. However, for the next few steps, you can disconnect them for convenience.

#### 5.2.2 Connect Data Logger with RTC Board to the PCB

**Fig. 22:**
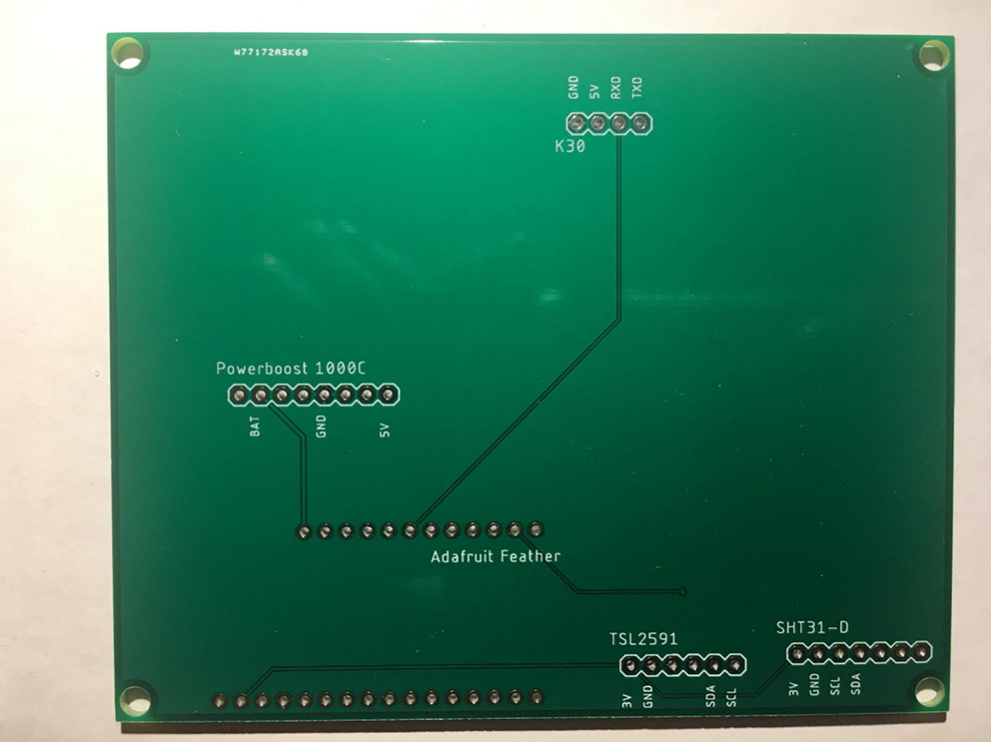
Top view of the eGreenhouse sensor PCB.

For this step, you need the PCB and Data Logger with RTC Board and the PCB. Fig. 22 shows the PCB front view. Make sure the PCB is facing forward.

**Fig. 23:**
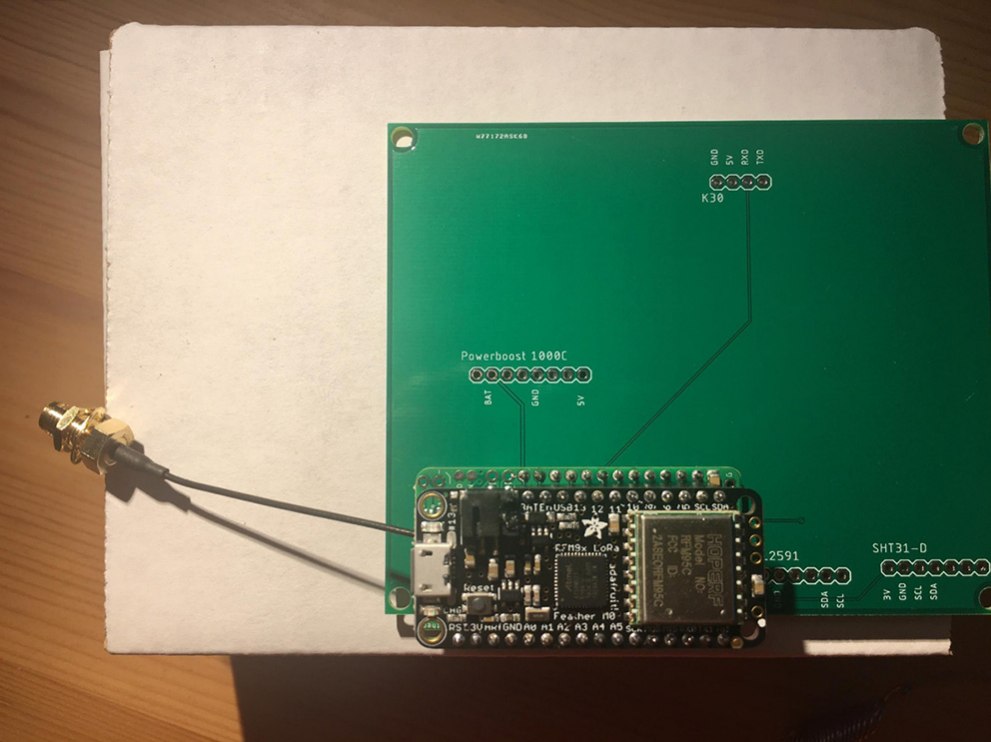
The top view of the PCB after soldering the Data Logger with the RTC Board and stacked Development Board.

**Fig. 24:**
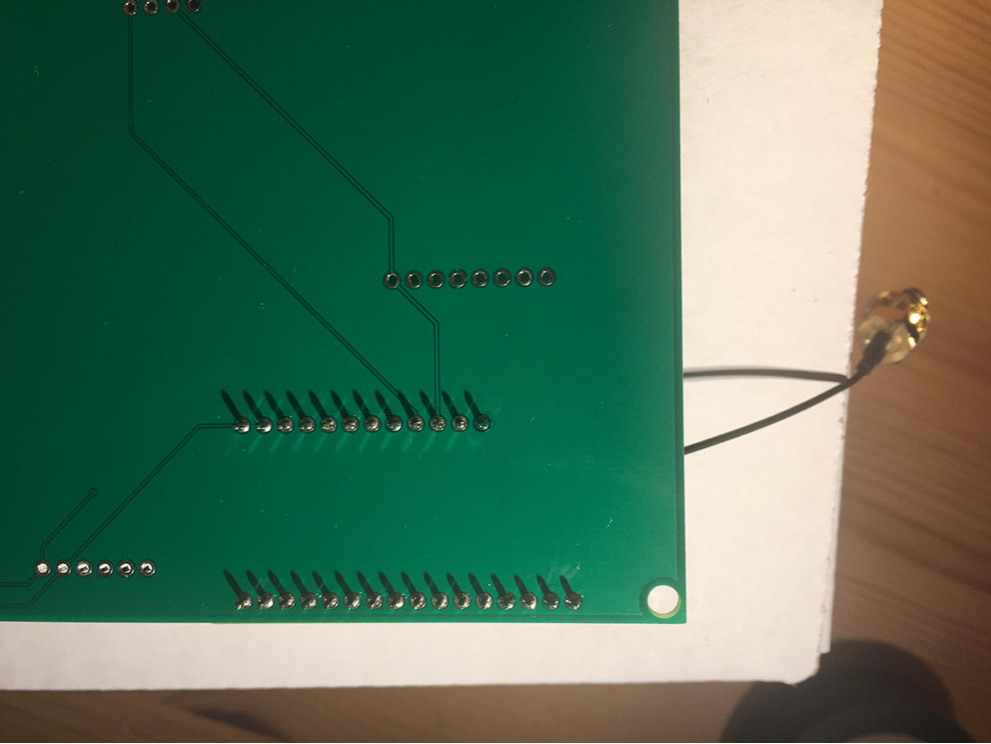
The bottom view of the PCB after soldering the Data Logger with the RTC Board.

Solder the Data Logger to RTC Board where it says “**Adafruit Feather**” on the PCB such that the long male headers are facing downward. Make sure the 12-pin male headers follows the 12-pin holes, the 16-pin male headers, and 16-pin holes. Fig. 23, Fig. 24 show both top and bottom view after soldering the Data Logger with RTC Board on the PCB. Alternatively, 12-pin and 16-pin female headers can be soldered onto the Sensor PCB to allow future removal of the Data Logger with RTC Board unless there is a height restriction.

#### 5.2.3 Connect Lux Sensor to the Sensor PCB

**Fig. 25:**
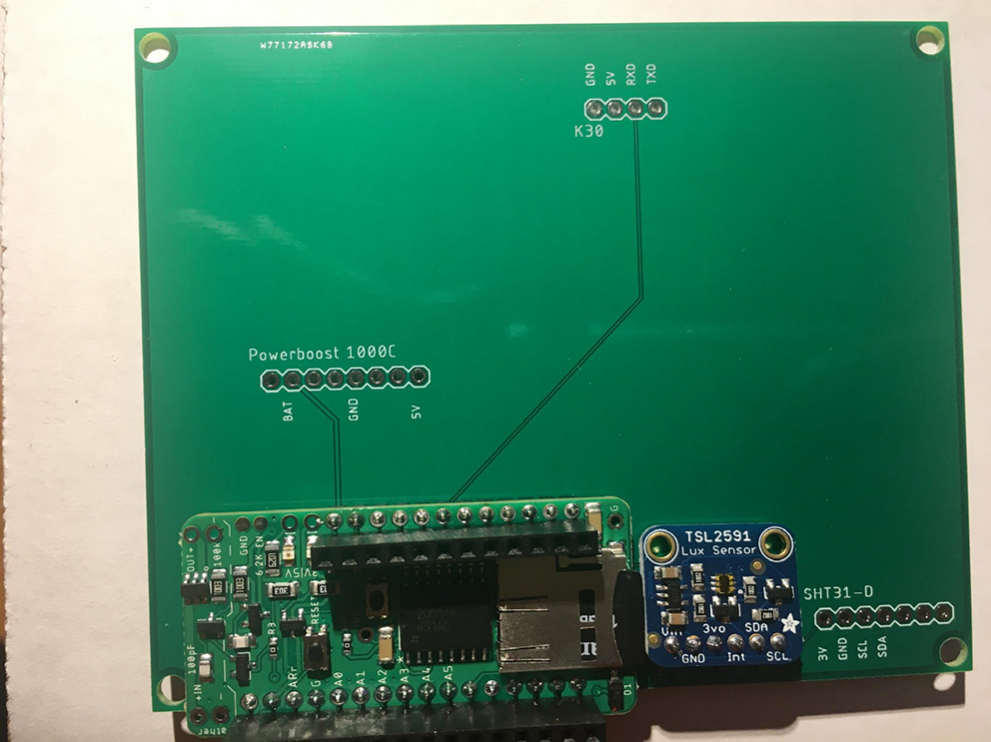
Top view after soldering the Lux Sensor to the PCB.

**Fig. 26:**
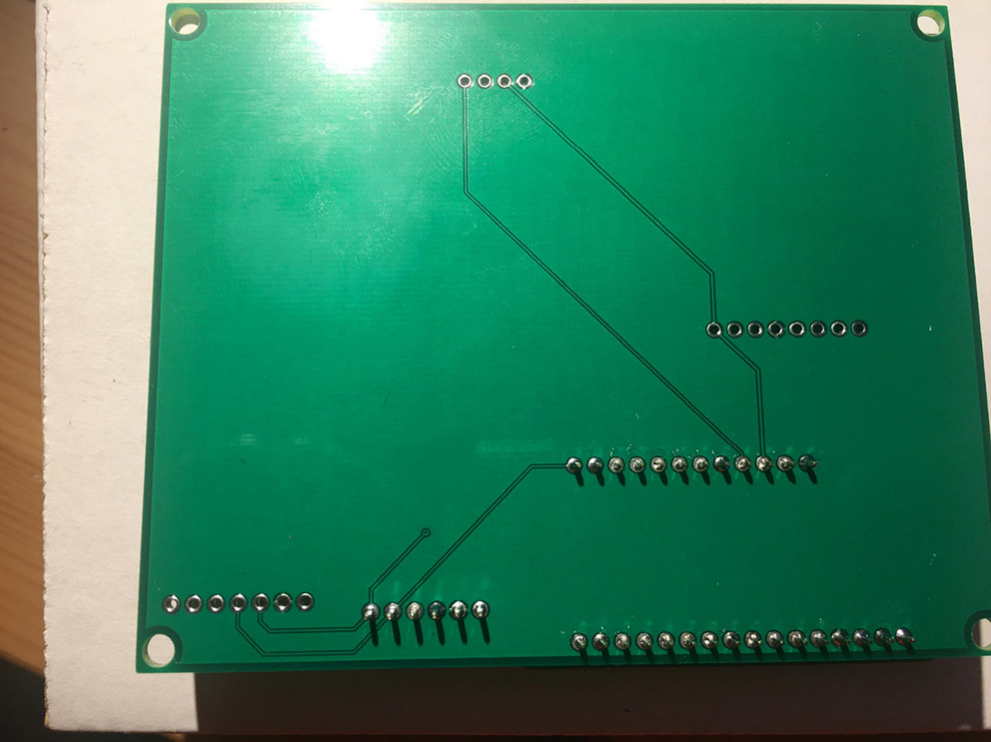
Bottom view after soldering the Lux Sensor to the PCB.

For this step, you need the Sensor PCB and Lux Sensor. Solder the Lux Sensor where it is labeled as “**TSL2591**”. Make sure that the label of each pin follows the correct pin hole. For example, the GND on the Lux Sensor is connected to GND on the PCB. Fig. 25, Fig. 26 show the top and bottom view after soldering Lux Sensor to the PCB.

#### 5.2.4 Connect Temperature and relative Humidity Sensor to the Sensor PCB

**Fig. 27:**
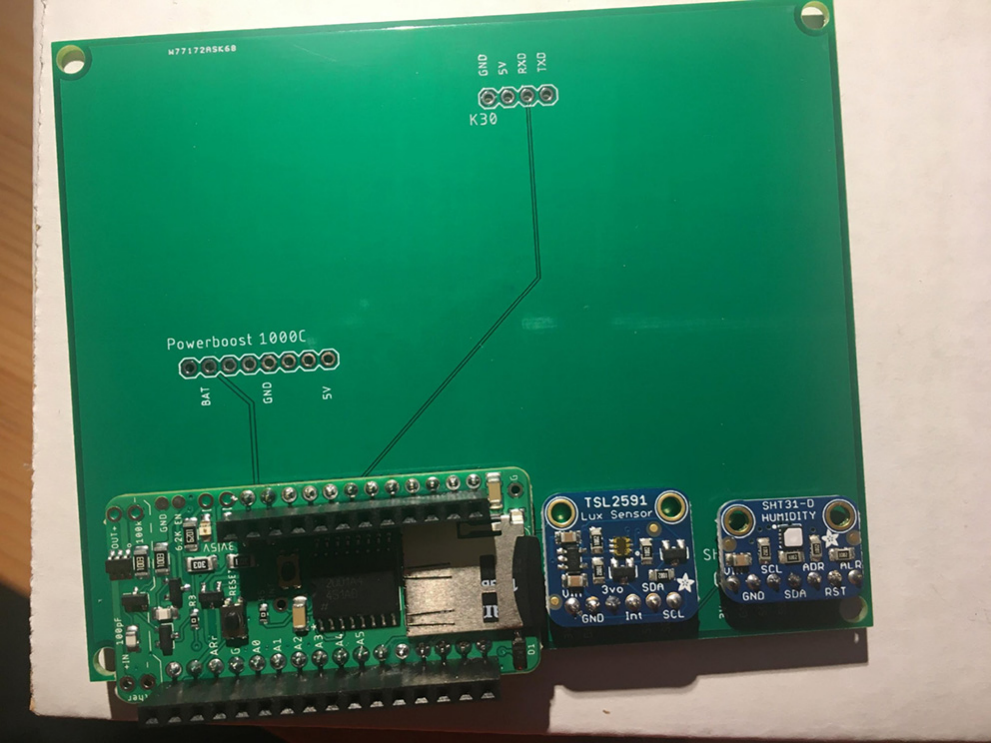
Top view after soldering the Temperature and Relative Humidity Sensor to the PCB.

**Fig. 28:**
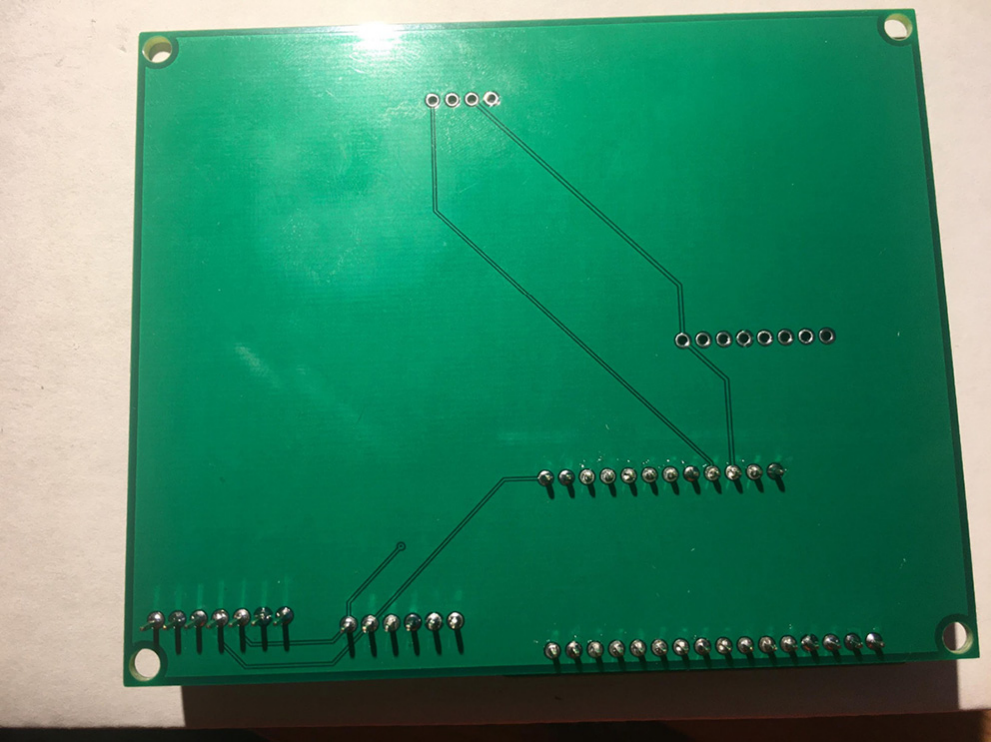
Bottom view after soldering the Temperature and Relative Humidity Sensor to the PCB.

For this step, you need the Sensor PCB and Temperature and Relative Humidity Sensor. Solder the Temperature and Relative Humidity Sensor where it is labeled as “**SHT31-D**”. Make sure that the label of each pin follows the correct pin hole. Fig. 27, Fig. 28 show the top and bottom view after soldering the Temperature and Relative Humidity Sensor to the PCB.

#### 5.2.5 Connect the PowerBoost to the Sensor PCB

**Fig. 29:**
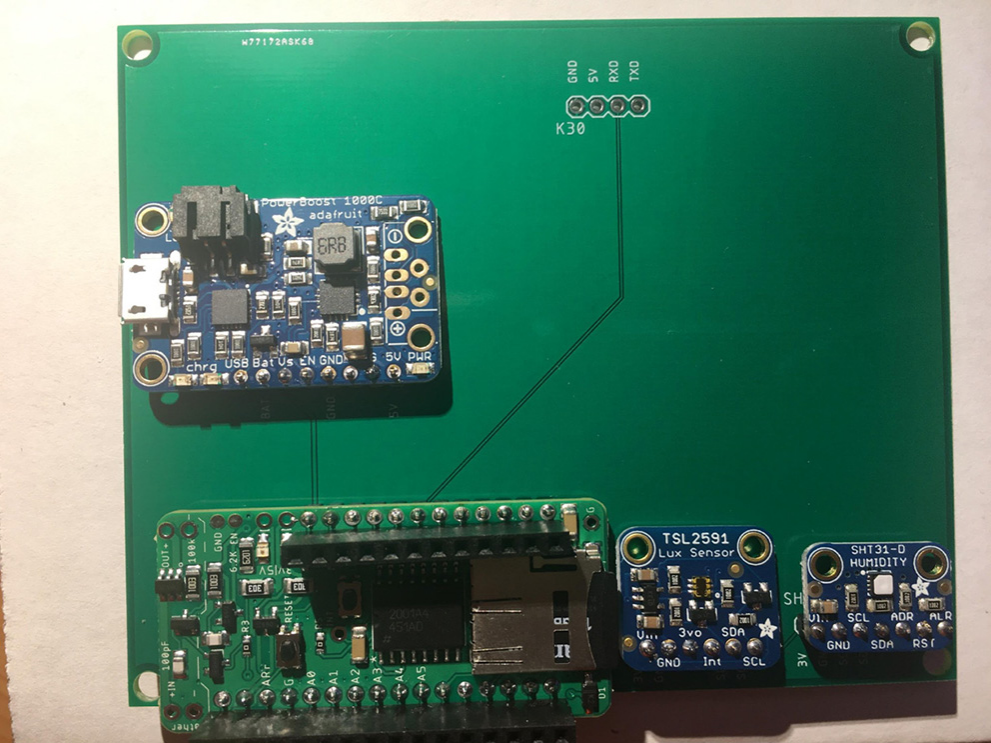
Top view after soldering the PowerBoost to the PCB.

**Fig. 30:**
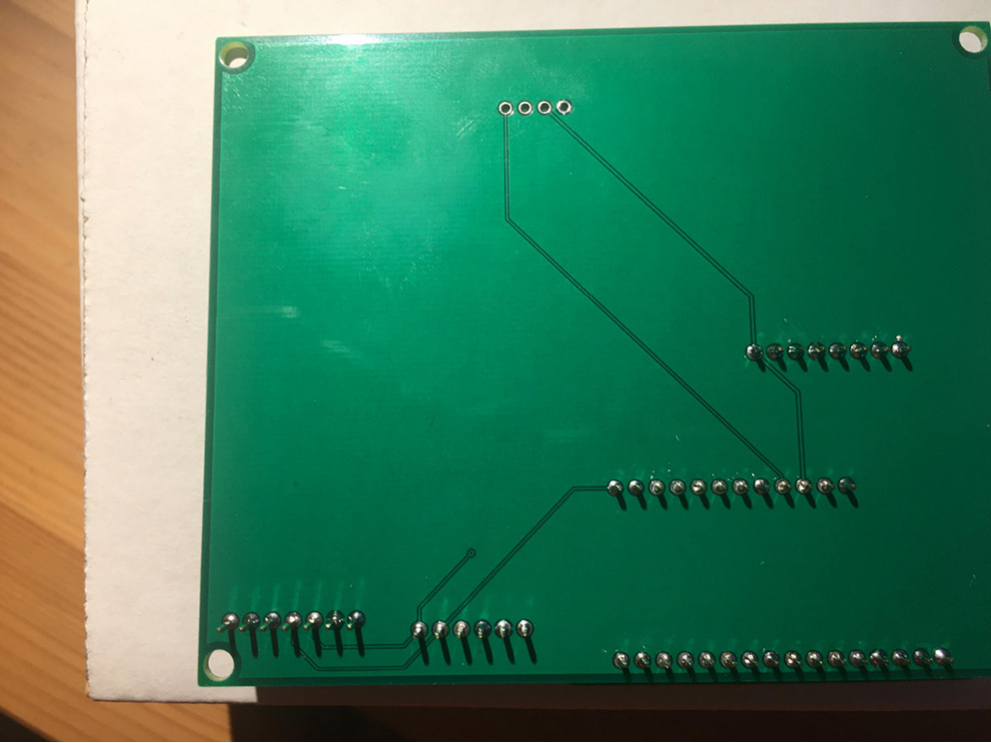
Bottom view after soldering the PowerBoost to the PCB.

For this step, you need PCB and PowerBoost. Solder the PowerBoost where it is labeled as “**PowerBoost 1000C.**” Make sure that the label of each pin follows the correct pin hole. Fig. 29, Fig. 30 show the top and bottom view after PowerBoost to the PCB.

#### 5.2.6 Connect CO_2_ Sensor to the Sensor PCB

**Fig. 31:**
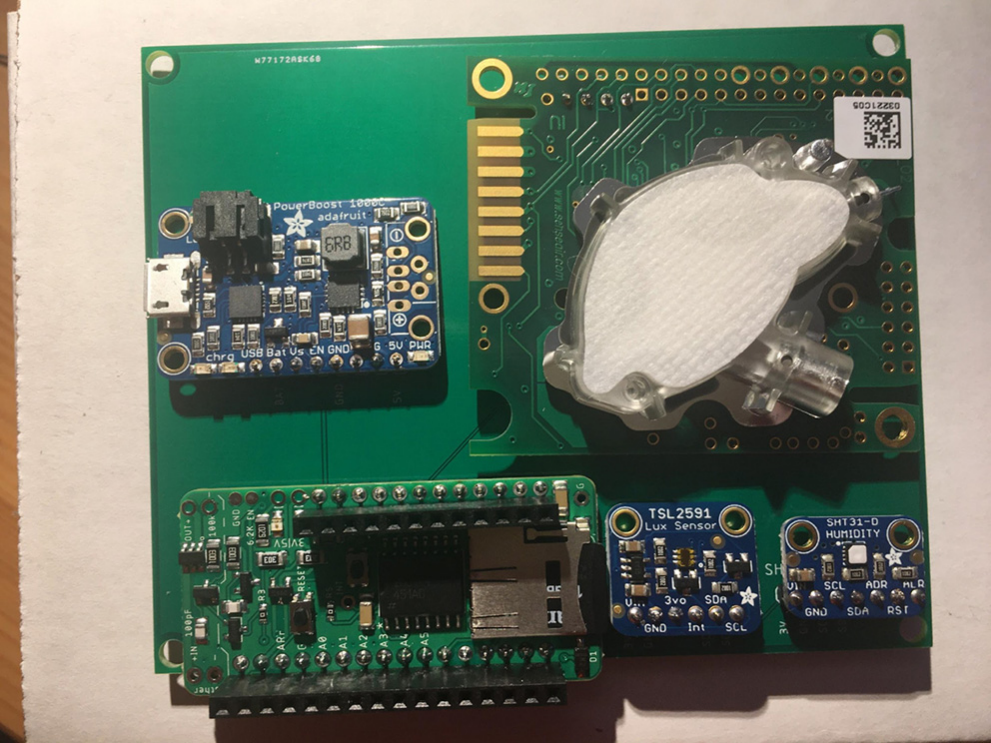
Top view after soldering the CO_2_ Sensor to the PCB.

**Fig. 32:**
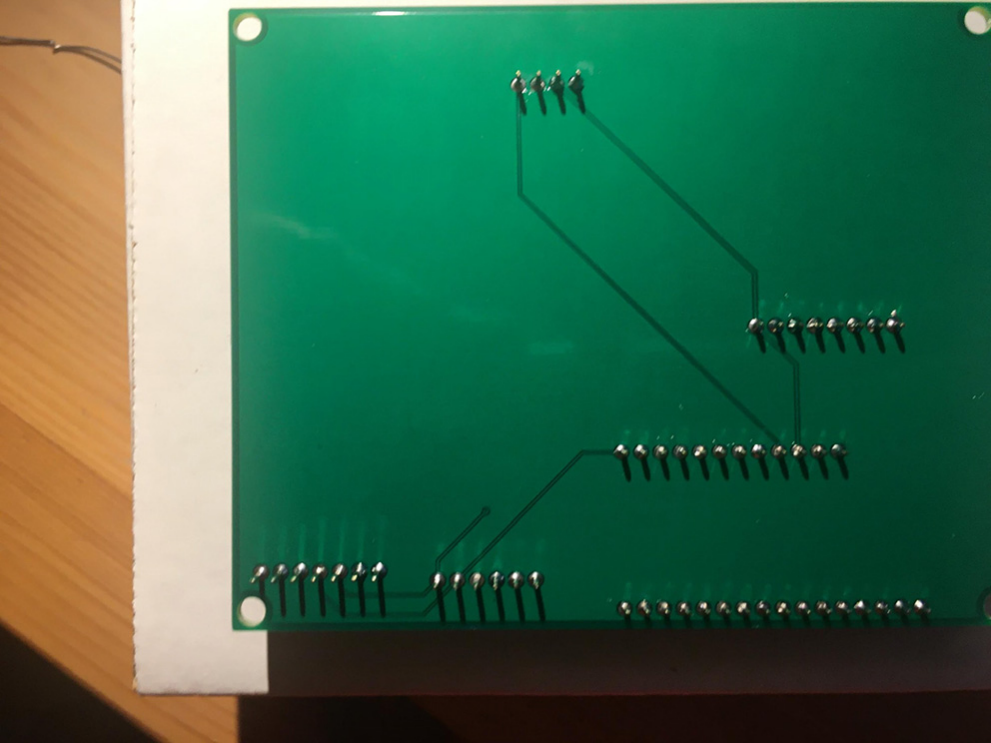
Bottom view after soldering the CO_2_ Sensor to the PCB.

For this step, you need the Sensor PCB and CO_2_ Sensor. Solder the CO_2_ Sensor where it is labeled as “**K30.**” Make sure to match the pin labels between the CO_2_ Sensor and Sensor PCB: G0 pin will be connected to GND on the PCB, G + to 5 V, RXD to RXD, and TXD to TXD. Fig. 31, Fig. 32 show the top and bottom view after CO_2_ Sensor to the Sensor PCB.

**Fig. 33:**
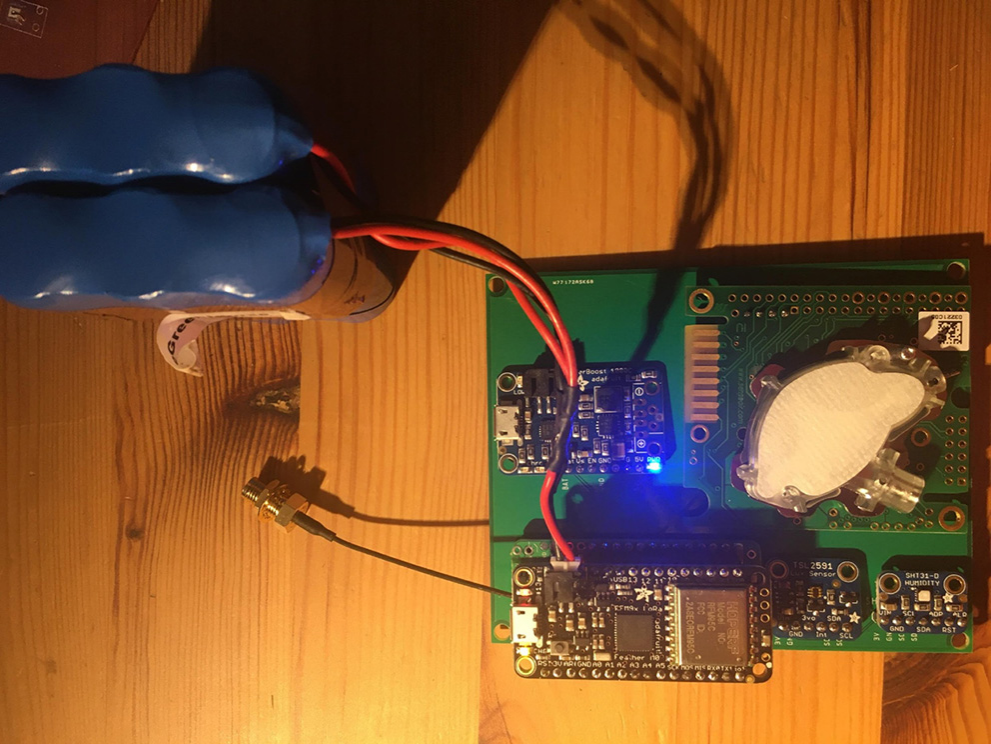
The eGreenhouse Sensor Package with power on from the battery.

Once that step is complete, you are done with the eGreenhouse Sensor Package Setup step. For verification, connect the battery to the Development Board as shown in Fig. 33**.** If it lights up for both the Data Logger with the RTC Board and the Development Board, and flashing for the CO_2_ Sensor, then everything is working perfectly.

### 5.3 Hub hardware setup

All steps in this section require a soldering iron and solder.

#### 5.3.1 Development Board

**Fig. 34:**
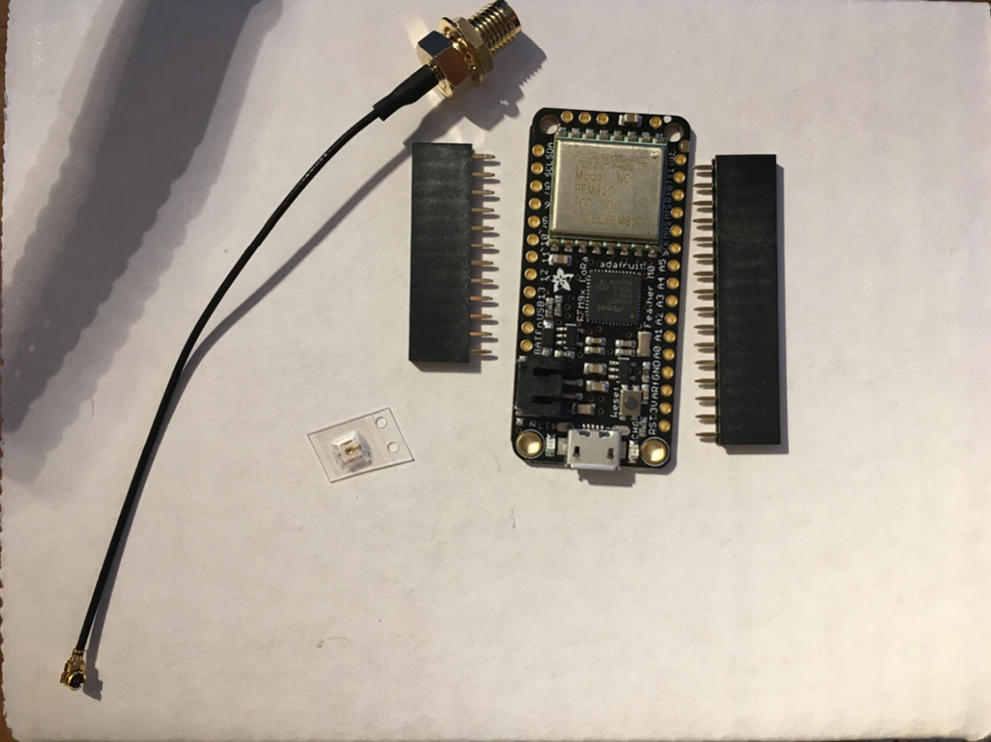
Antenna Connector, Antenna Kit, Development Board, and 12-pin + 16-pin female headers for hub setup.

In this step, you need the Antenna Connector, Antenna Kit, Development Board, and 12-pin + 16-pin female headers (Fig. 34).

**Fig. 35:**
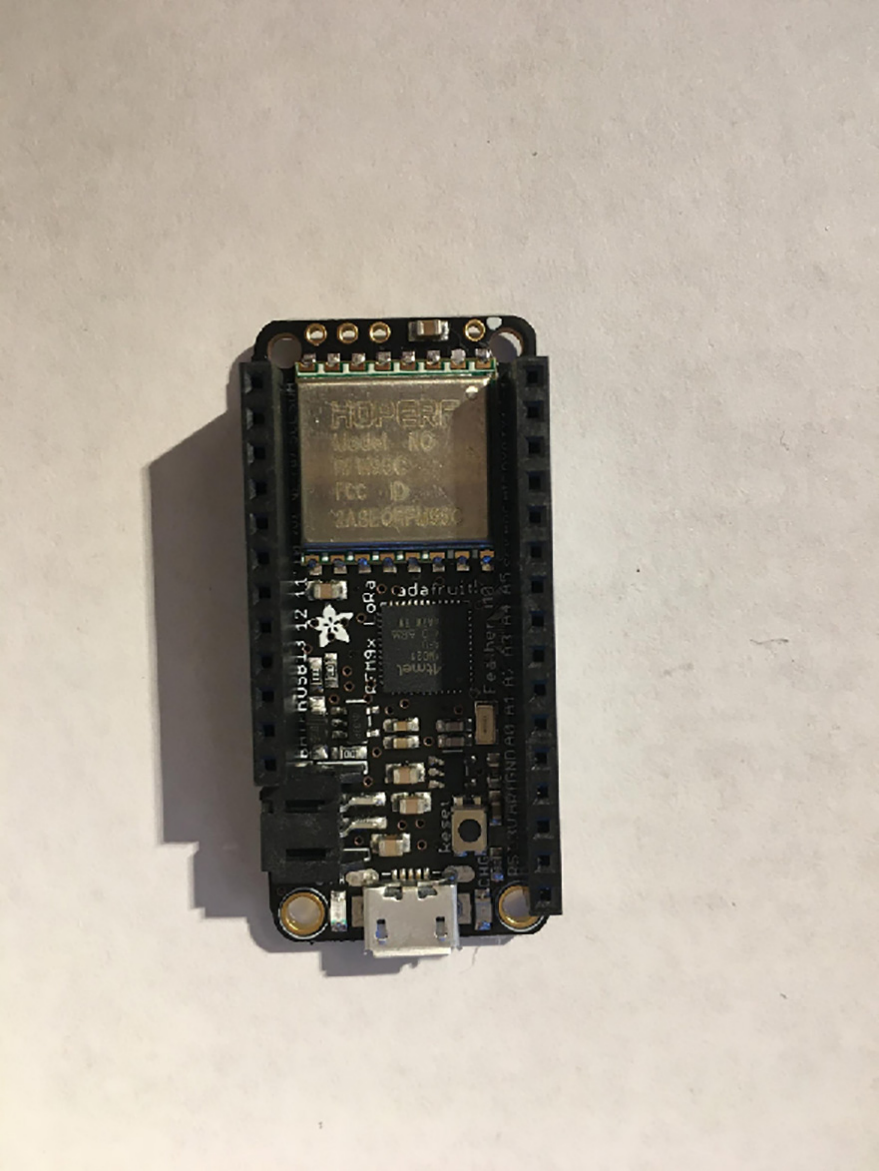
Top view after soldering female headers on the Development Board.

**Fig. 36:**
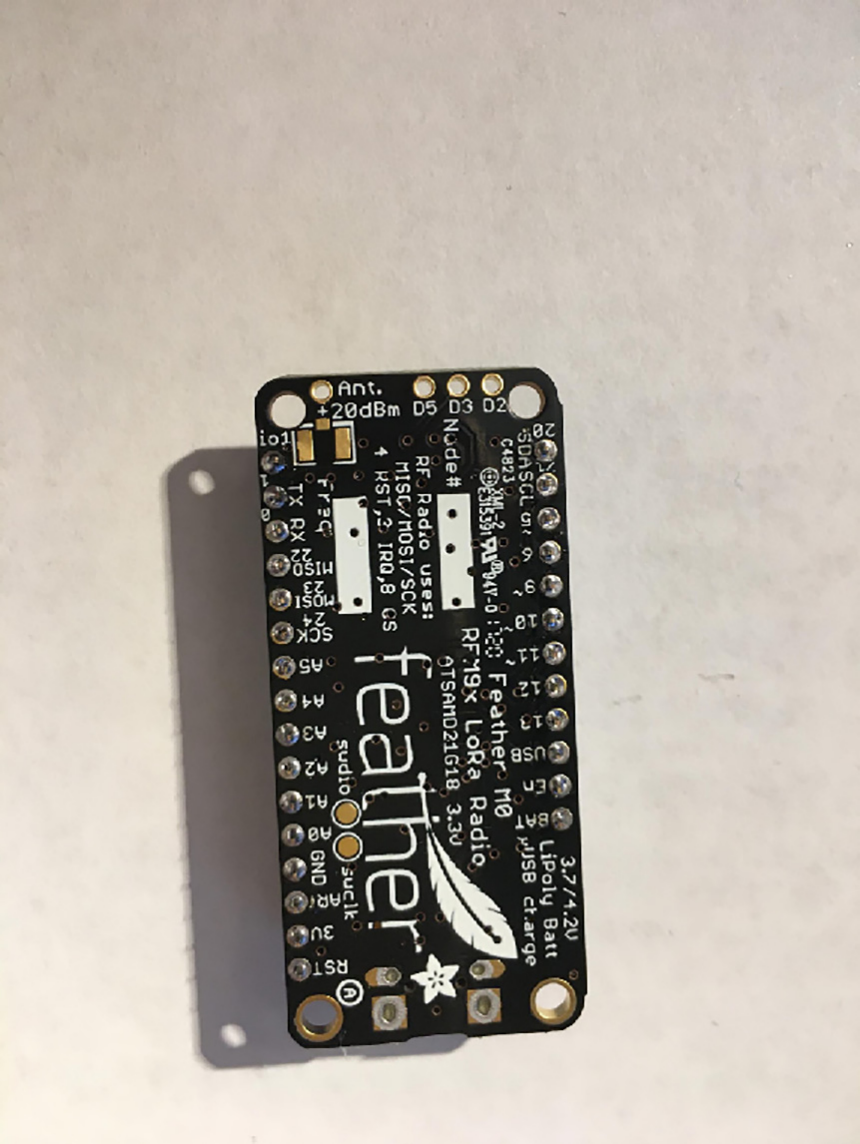
Bottom view after soldering female headers on the Development Board.

First solder 12-pin + 16-pin female headers to the Development Board such that the socket side is on the top of the board (Fig. 35, Fig. 36).

**Fig. 37:**
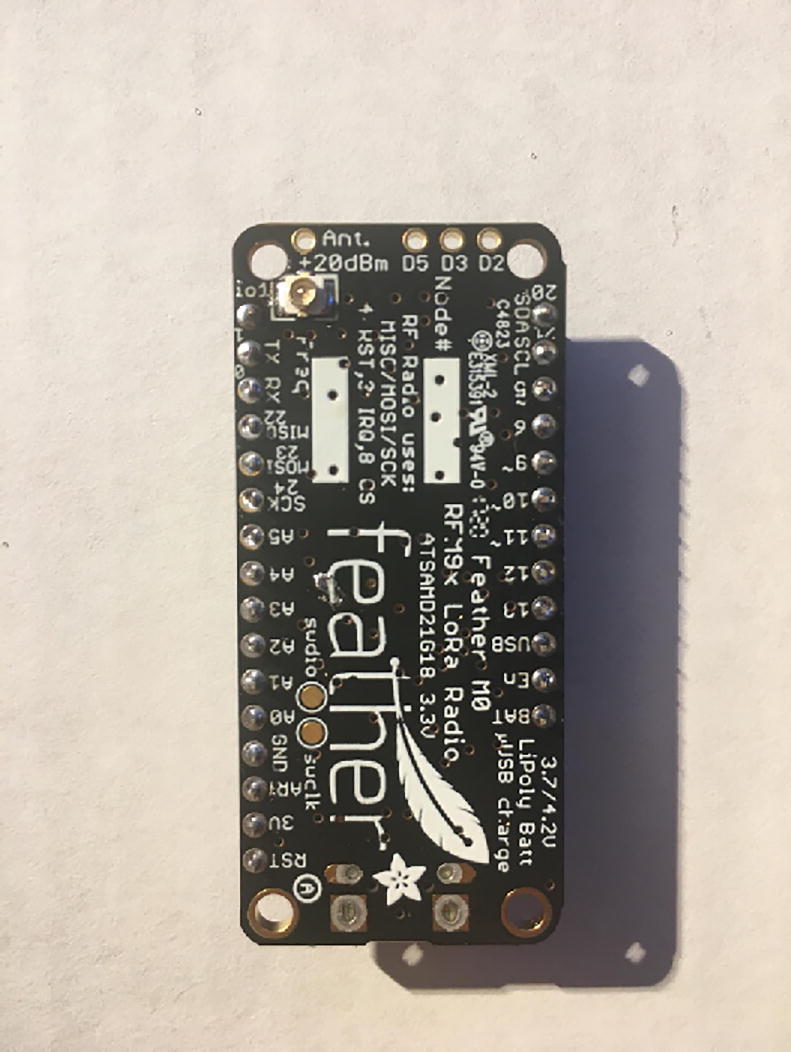
Bottom side of the Development Board for the hub after soldering on the Antenna Connector.

Once that step is complete, then solder the Antenna Connector to the bottom side of the Development Board (Fig. 37).

**Fig. 38:**
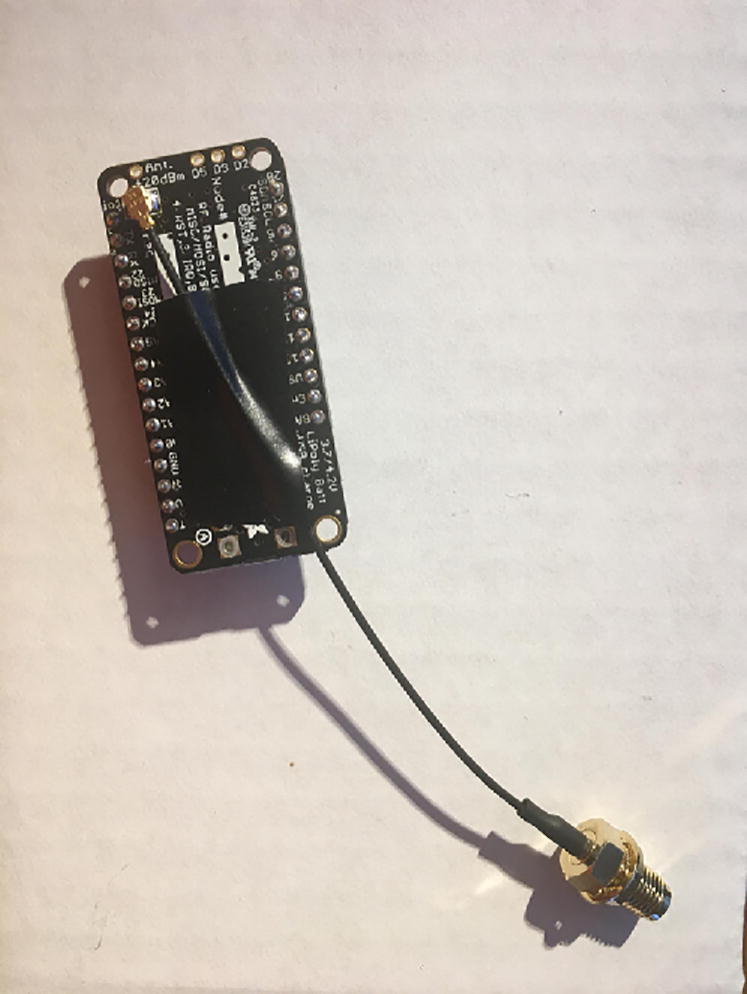
The Development Board for the Hub.

Once that step is complete, then connect the Antenna Kit, except the actual plastic antenna, on the Antenna Connector. Because it is easy to be disconnect, either add electrical tape or hot glue so that it will be in position (Fig. 38).

#### 5.3.2 Ethernet Connector

**Fig. 39:**
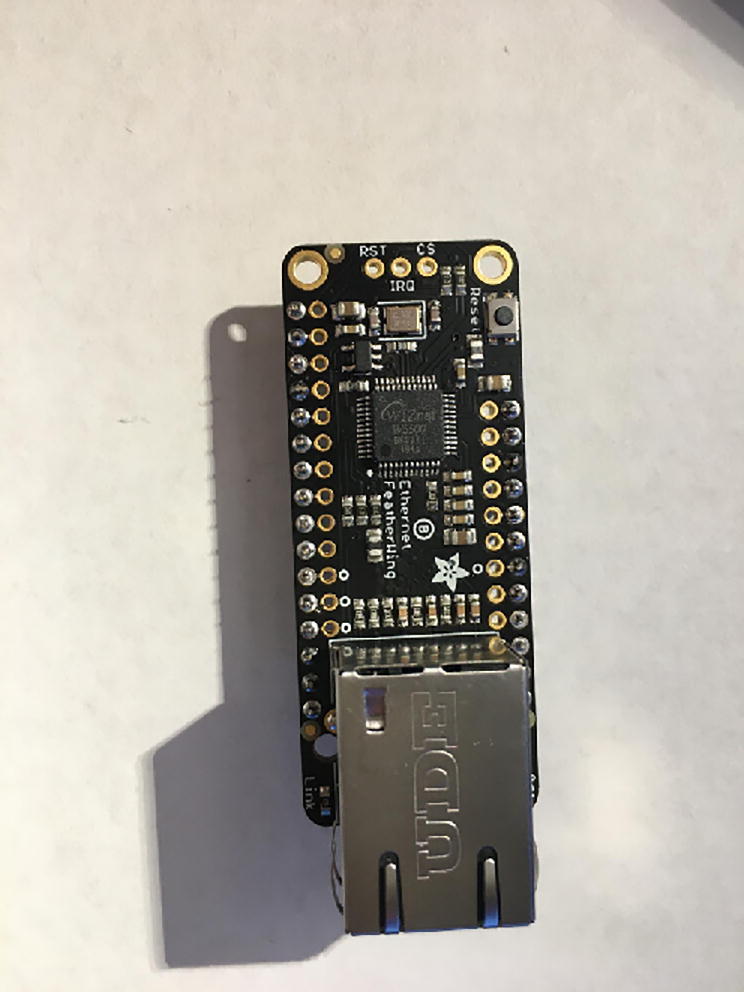
Top view after soldering the male headers on the Ethernet Connector.

**Fig. 40:**
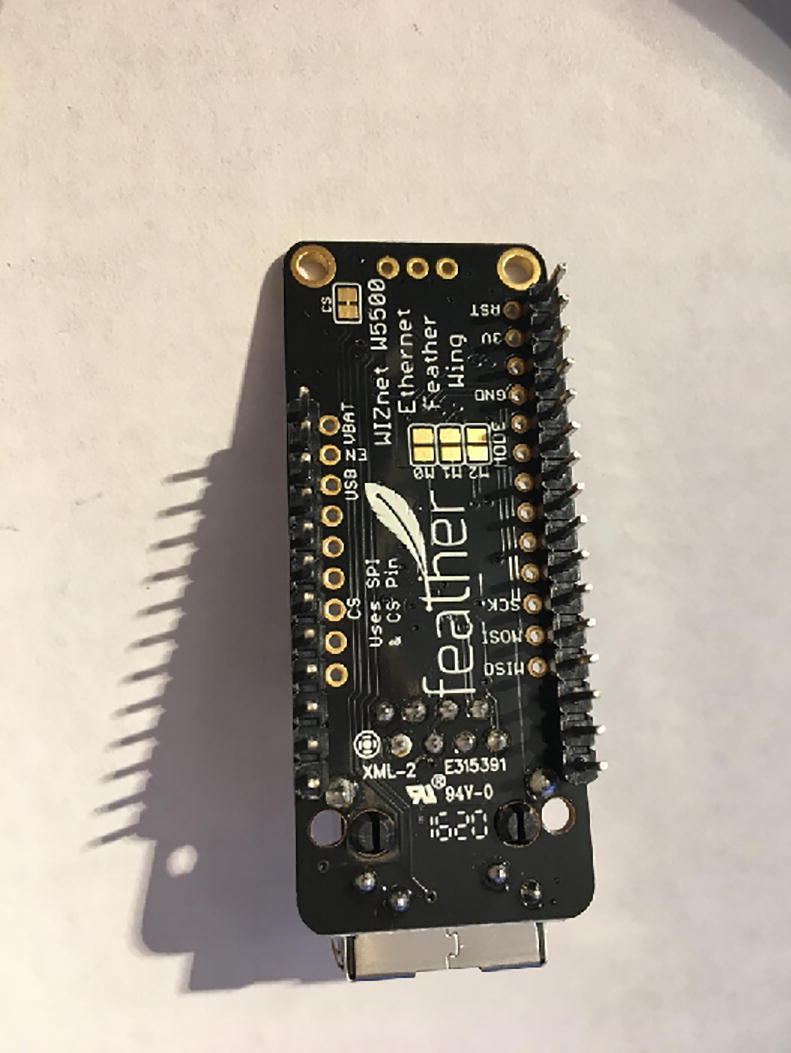
Bottom view after soldering the male headers on the Ethernet Connector.

For this step, you need Ethernet Connector and 12-pin + 16-pin male headers that came with the Development Board package. Solder the male headers facing down (Fig. 39, Fig. 40).

#### 5.3.3 Connect Ethernet Connector to Development Board

**Fig. 41:**
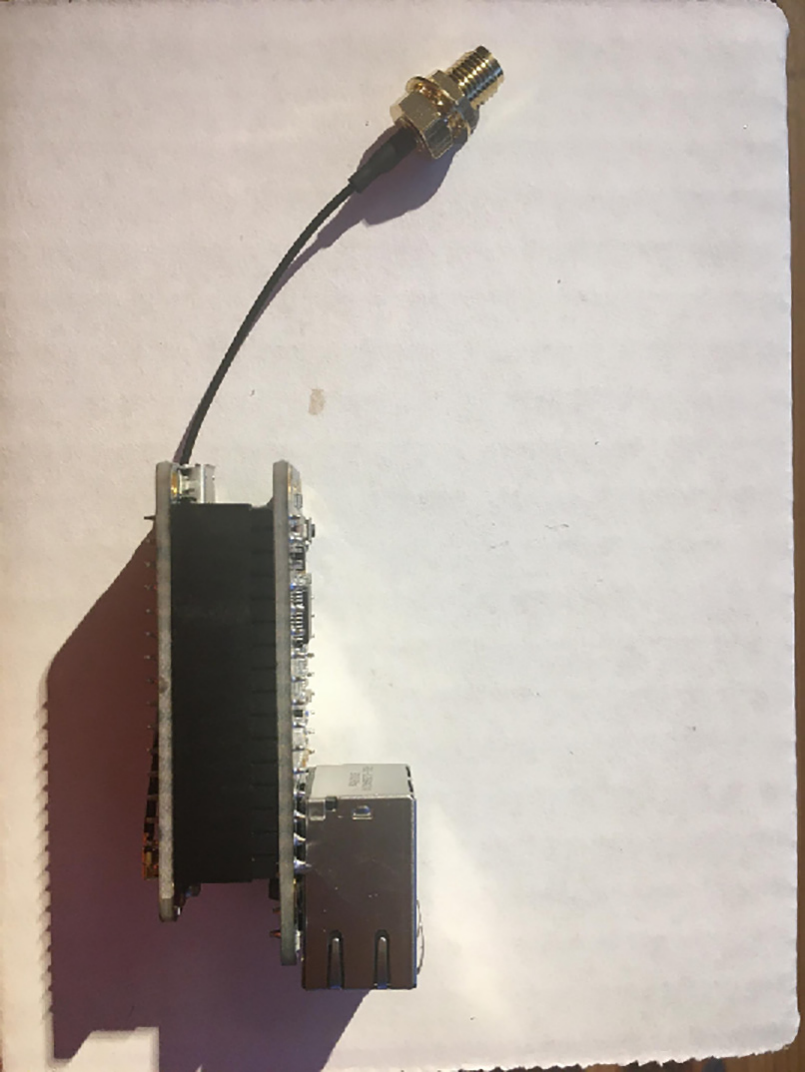
The connected Development Board and Ethernet Connector.

For this step, you need the Development Board and Ethernet Connector. Connect the Ethernet Connector on top of the Development Board (Fig. 41).

Once that is done, you are completely done with the Build Instructions.

## 6 Operation instructions

### 6.1 Software download

If you are using the Windows Operating System, download Git Bash Shell, Arduino IDE, and Processing.

If you are using the Mac Operating System, download Arduino IDE and Processing. You do not need Git Bash Shell.

Open Git Bash for Windows and Terminal for Mac. Enter the following command. This should install all the dependencies and libraries to operate properly.

curl -fsSL https://raw.githubusercontent.com/OPEnSLab-OSU/Loom/master/setup/setup-windows.sh | sh

**Fig. 42:**
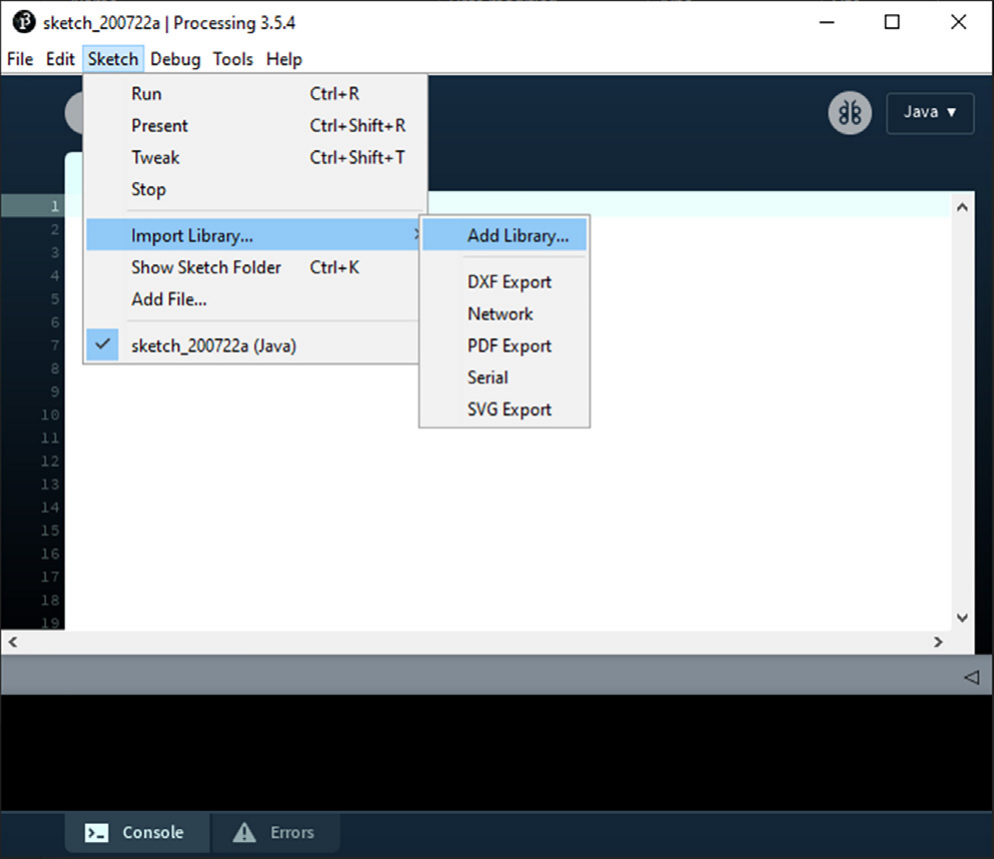
Processing Application: where to select to add libraries to the board.

Upon completion, open Processing. Then go to Sketch -> Import Library… -> Add Library… See Fig. 42 for location of the option.

**Fig. 43:**
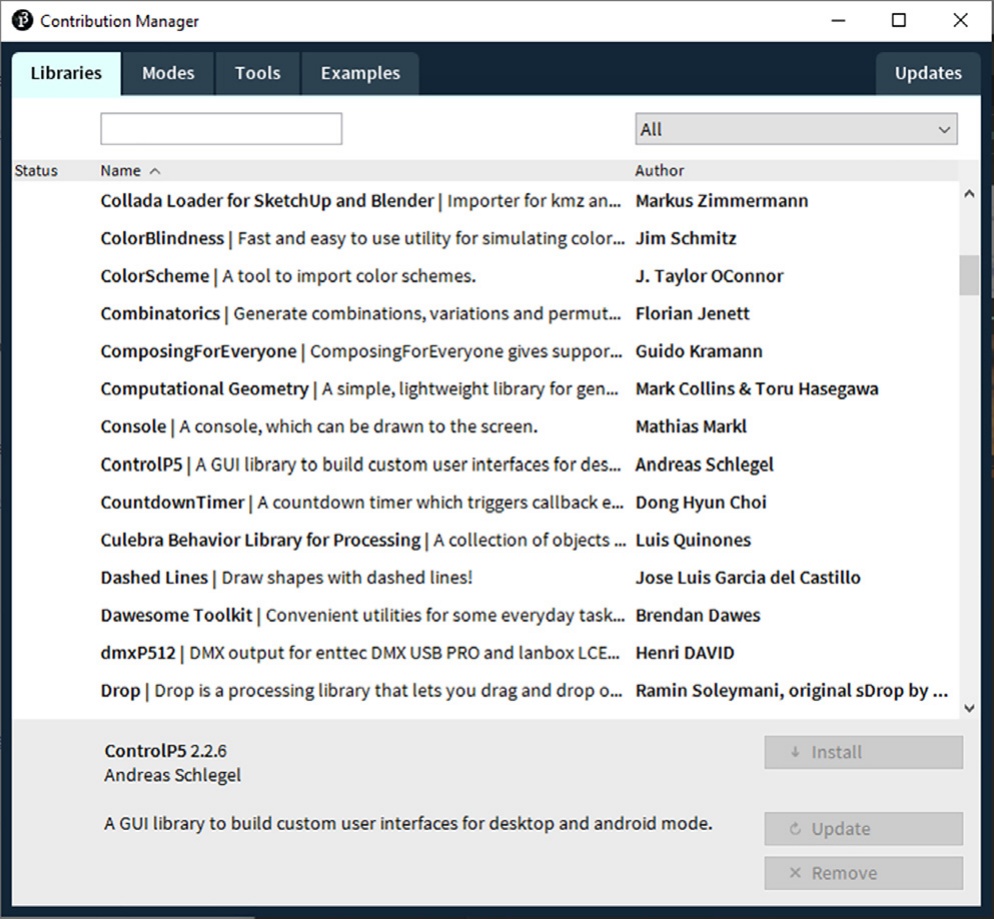
The Processing Contribution Manager for installing new Libraries for the Processing.

Once you select Add Library, you will get a pop shown in Fig. 43. From the menus on the top left of the window, search both Control5P and Arduino (Firmate) and install both.

**Fig. 44:**
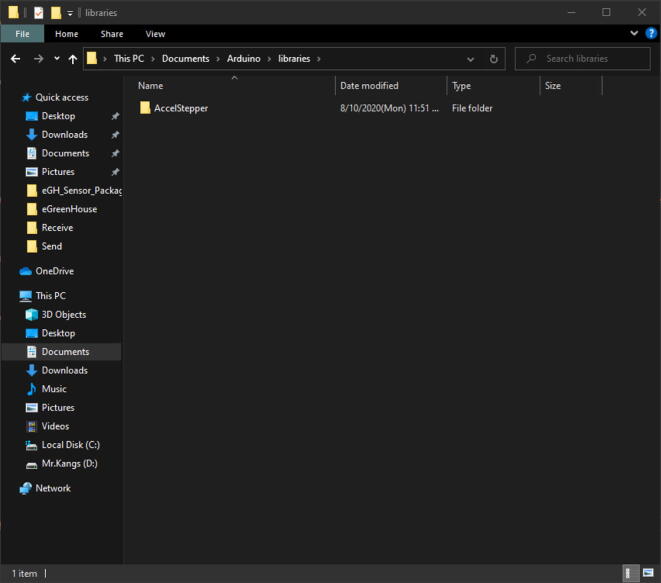
Paste the AccelStepper folder in Document -> Arduino -> libraries. Here, the libraries folder is located This PC -> Document -> Arduino -> libraries.

In the Code folder, which is under the Design folder, you can find a folder named Dependencies. In that folder, copy the AccelStepper folder and paste it in Document -> Arduino -> libraries such as Fig. 44. Each system setup might vary the location of the folder; therefore, please check where the Arduino -> libraries is located.

Once you complete this setup, then you are set up with the Arduino IDE software.

**Fig. 45:**
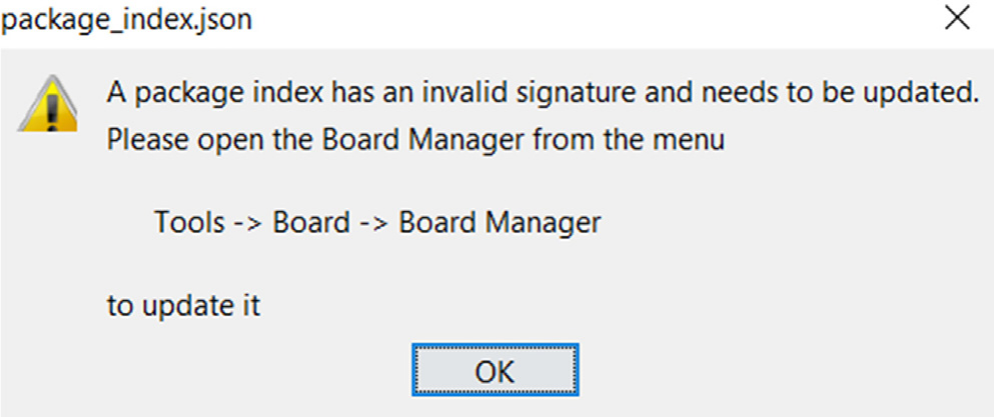
Warning message after installing the Arduino and the Command line. This can be ignored.

Note: when you open the Arduino IDE, you will get the following message in Fig. 45. You can simply ignore that.

### 6.2 eGreenhouse Sensor package

There are two different versions of eGH_Sensor_Package code to use.

If you are using Adalogger from Adafruit as the Data Logger with the RTC Board, then open the folder called **Non Hypnos eGH_Sensor_Package**.

If you follow the build instructions using the OPEnS Data Logger with RTC Board, then open the folder called **Hypnos eGH_Sensor_Package**.

**Fig. 46:**
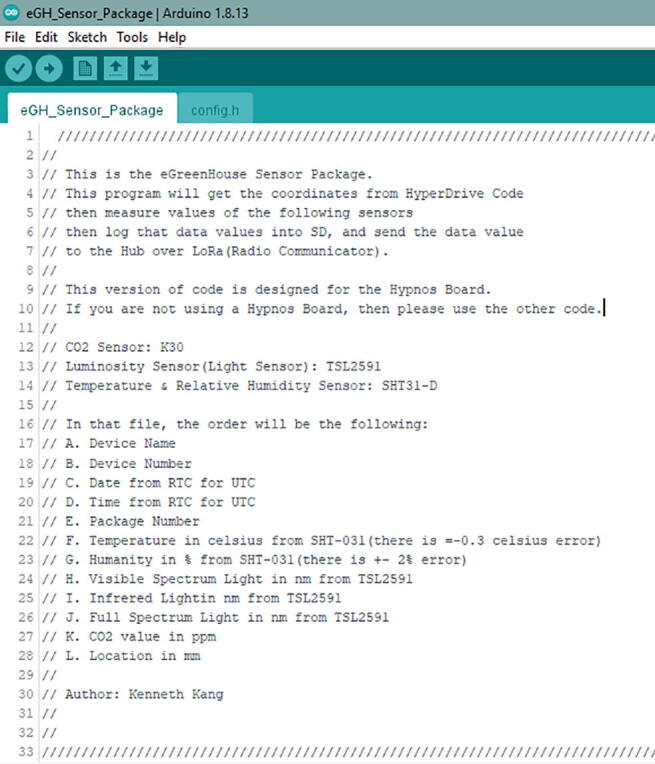
The eGH_Sensor_Package.ino code from the Arduino IDE. Read the description for version type and additional information.

In the code folder, go to **Hypnos eGH_Sensor_Package** folder. Then inside that folder there will be another folder called eGH_Sensor_Package. In that folder, open eGH_Sensor_Package.ino file, and you will see code as in Fig. 46**.**

**Fig. 47:**
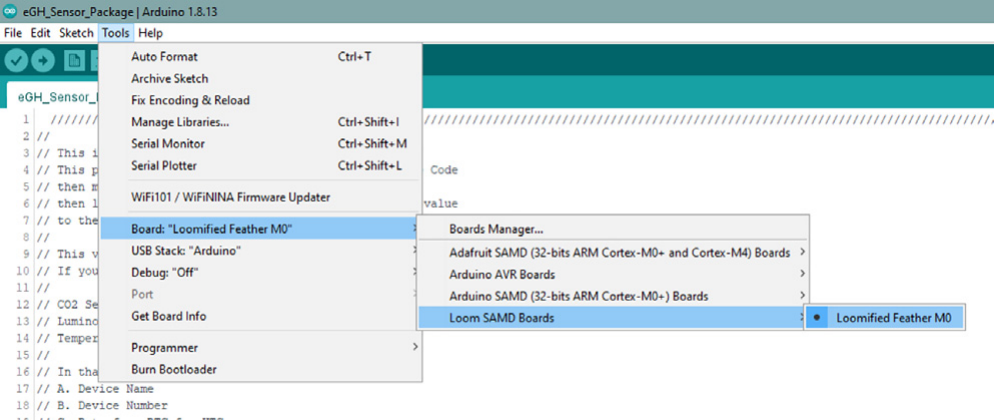
Selecting the Board in the Arduino IDE.

In that screen, select Tools -> Board -> Loom SAMD Boards -> Loomified Feather M0 (Fig. 47).

**Fig. 48:**
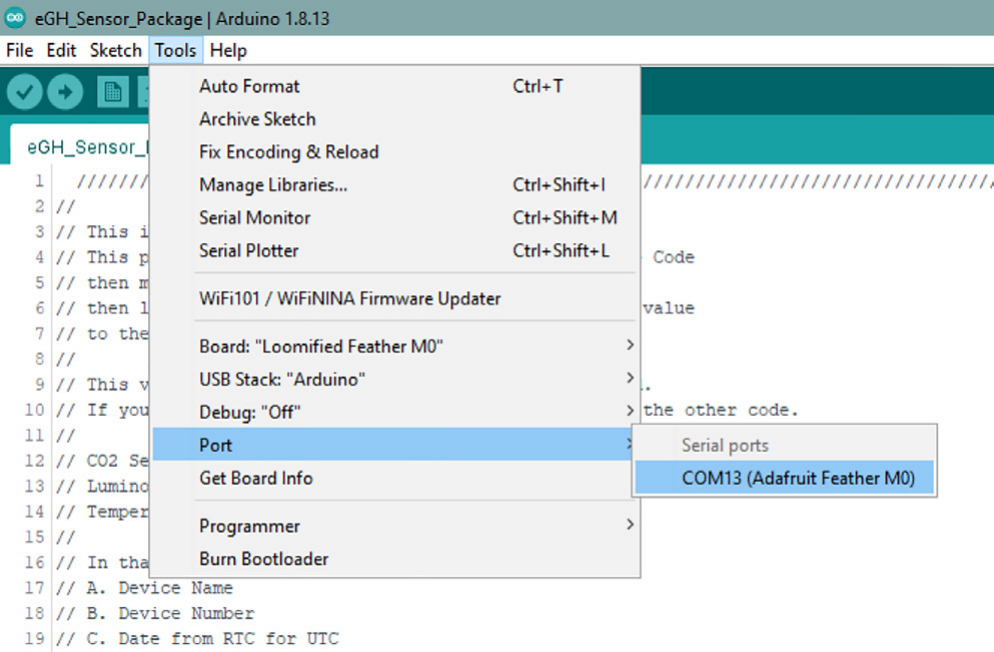
The proper look when you connect the eGreenhouse Sensor Package. Note that the port number may be different.

Once that is complete, connect the eGreenhouse Sensor Package to the computer with Micro USB Cable. The Micro USB Cable should be connected to the Development Board, not the PowerBoost. It should recognize the board. Then go to Tools -> Port and see if there is an option to select. If there is no option, try either a different cable or reconnect it. It should be as in Fig. 48.

Once the computer recognizes the board, then go to the config.h. In that file, you will find either

{'name':'DS3231′,'params':[10,false]}

or

{'name':'PCF8523′,'params':[10,false]}

In the config, you can see it has the same as [10,false]. The 10 is indicating the time zone, which it is currently set up as PDT. You can change the time zone by referring here. For example, if you are in Eastern Standard Time, it will be 5 rather than 10, which will be [5,false].

**Fig. 49:**
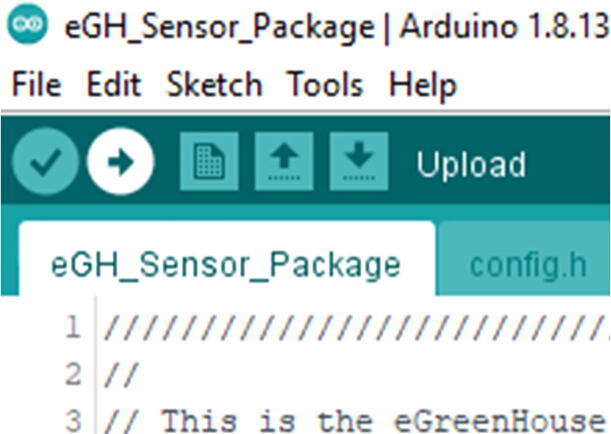
Upload button on the Arduino IDE.

Once you change the config.h file, then click upload using the arrow pointing to the right (Fig. 49).

**Fig. 50:**
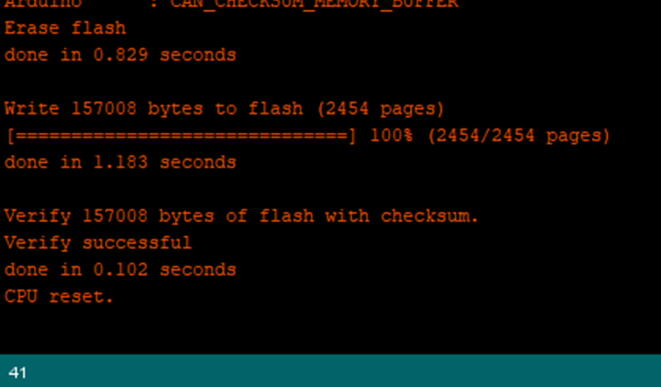
The console log showing successful upload.

Wait until it gets the message shown in Fig. 50. Once the message appears as “CPU Reset”, then it has uploaded properly, and is ready to use.

### 6.3 Hub

#### 6.3.1 Before for setting up the Hub

For this step, you will need Ethernet cable that must be connected to an open Ethernet port. At the same time, you will need your own Mac Address. Make sure the Mac Address is in decimal (not hexadecimal).

#### 6.3.2 GoogleSheets setup

**Fig. 51:**
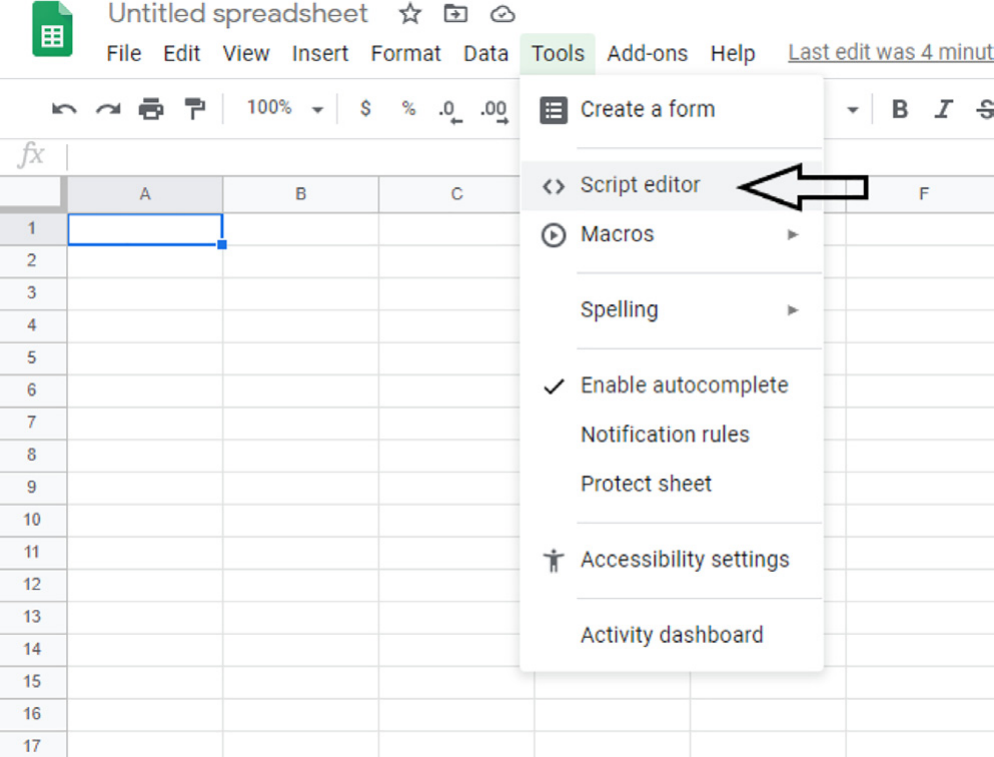
The drop-down menu location where Script editor is found.

**Fig. 52:**
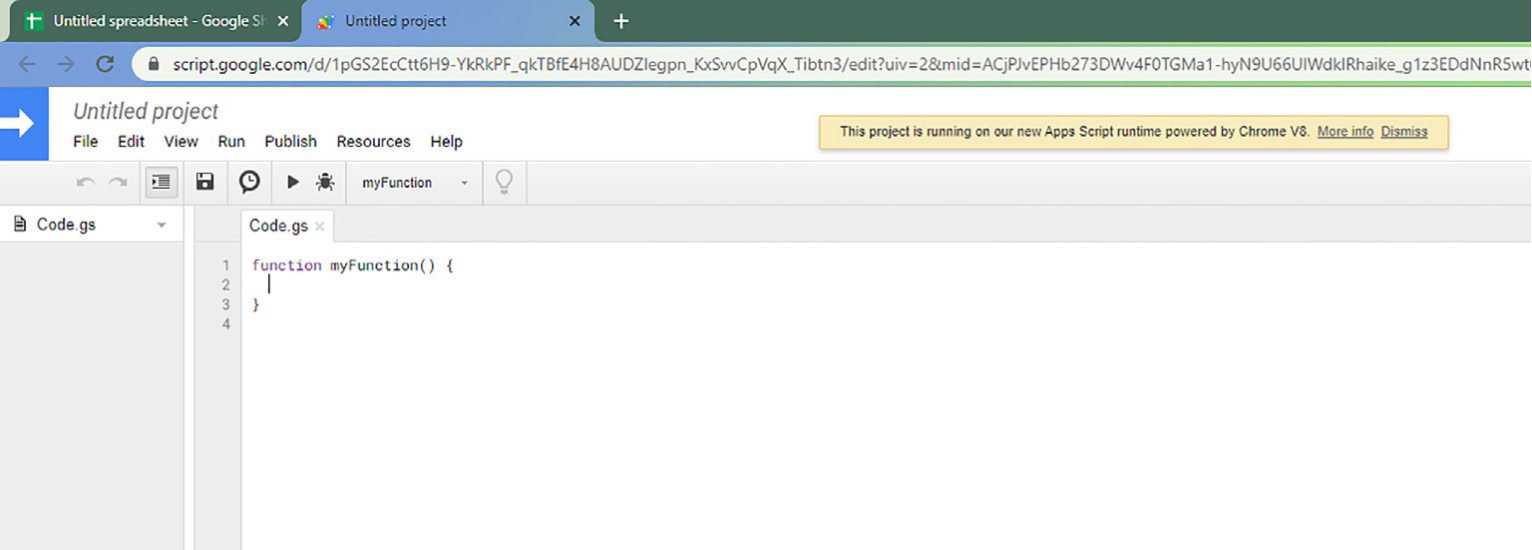
The Script editor tab for your GoogleSheets.

First, create a new GoogleSheets in either your personal drive or an organization drive. Once it is created, go to Tools -> Script editor (Fig. 51). Once you select Script editor, then you will get a new tab (Fig. 52).

**Fig. 53:**
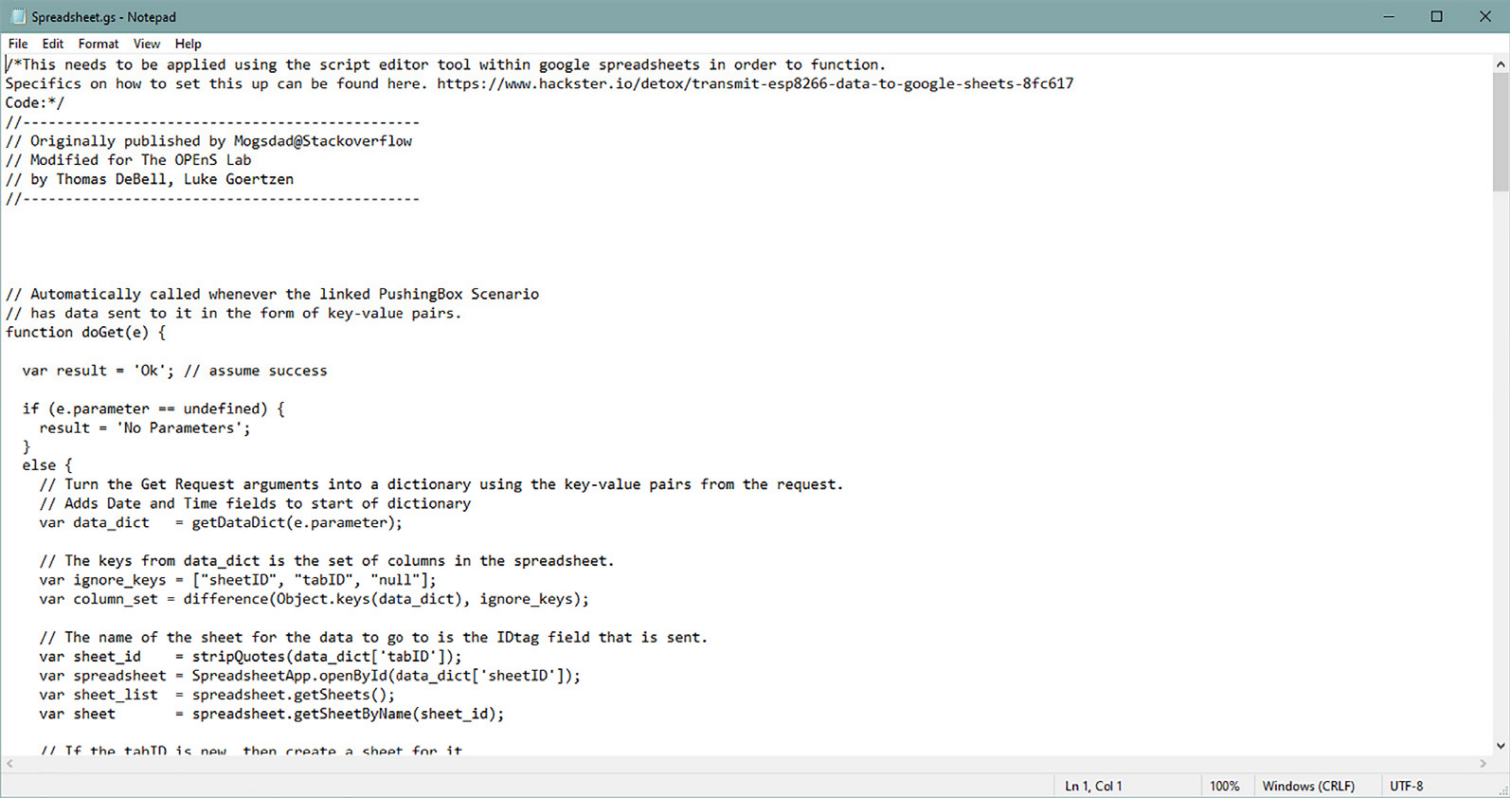
The Spreadsheet.gs file opened with Notepad.

Inside the Code file, go to the folder called **GoogleSheets**. Inside, you will have Spreadsheet.gs. Open that file with any text editor or simply notepad (Fig. 53)

**Fig. 54:**
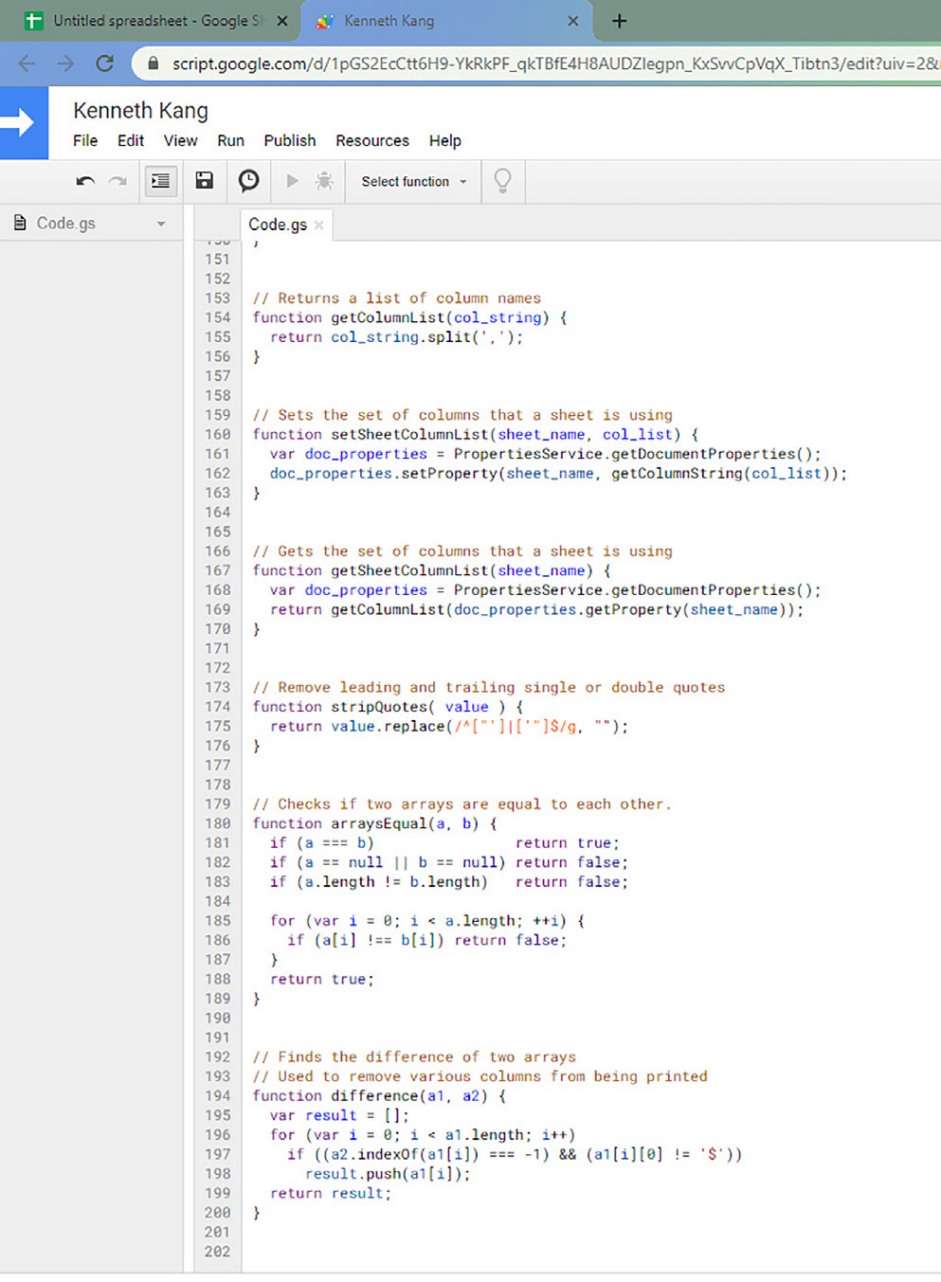
The Spreadsheet.gs code on your Script editor.

In this file, copy everything and paste that in the Script editor. Before you paste it to the Script editor, make sure it is empty (Fig. 54).

**Fig. 55:**
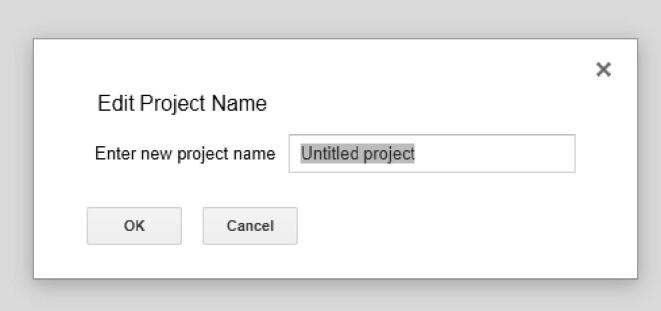
Pop up for saving the Script Editor.

Once you save it, you will get this pop up (Fig. 55).

**Fig. 56:**
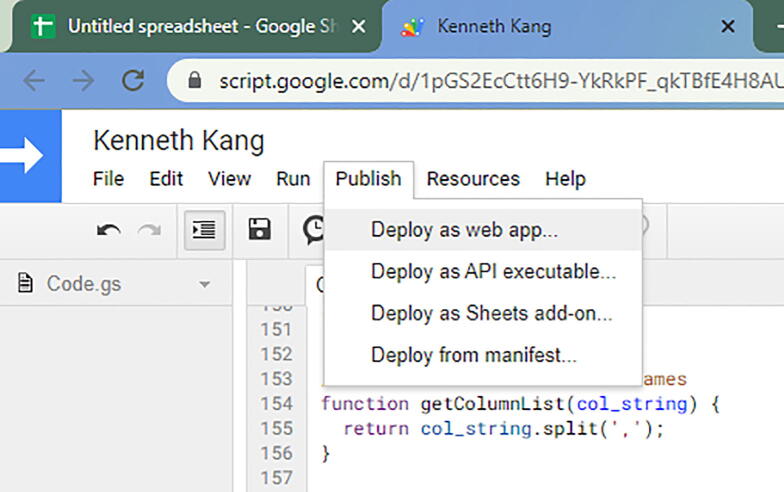
The option to select Deploy as web app…

You can name the project as you prefer. Once that is complete, then go to Publish -> Deploy as web app… (Fig. 56).

**Fig. 57:**
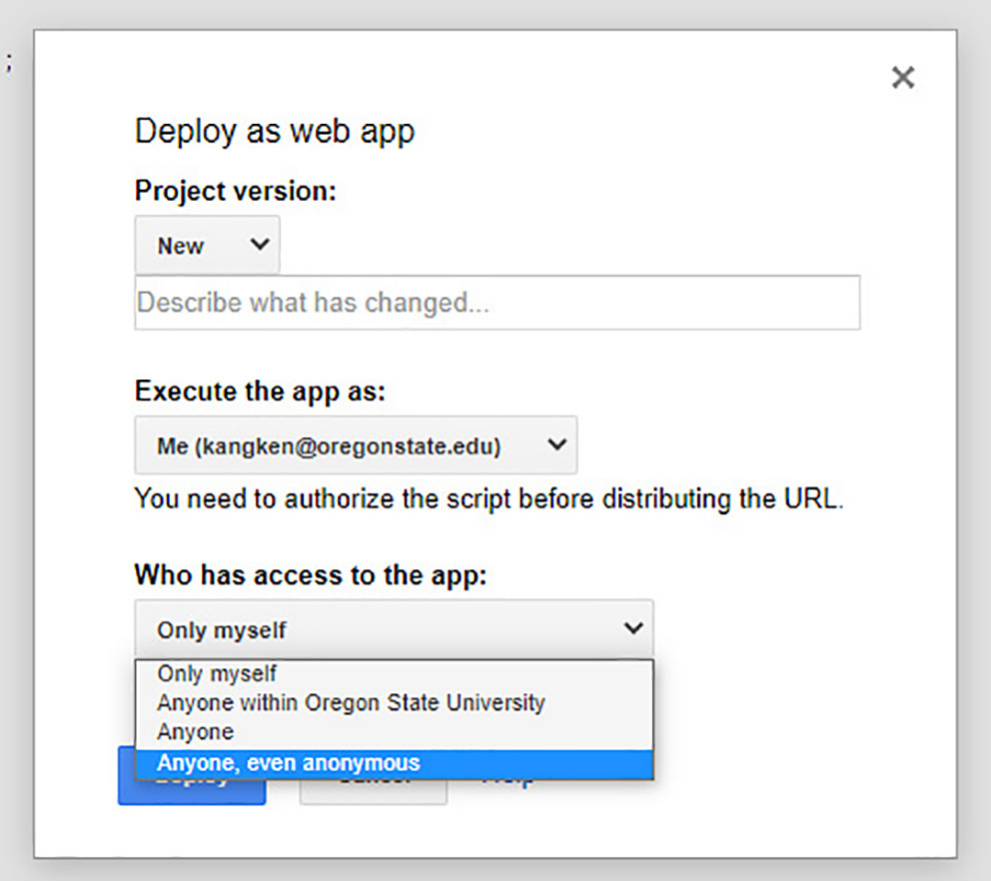
The option to select Anyone, even anonymous rather than Only myself.

In there, change the option Who has access to the app from Only myself to Anyone, even anonymous. (Fig. 57).

Once you change the option, then select the blue button Deploy. There will be some permission review when you select that option. Make sure you authorize with your google account.

**Fig. 58:**
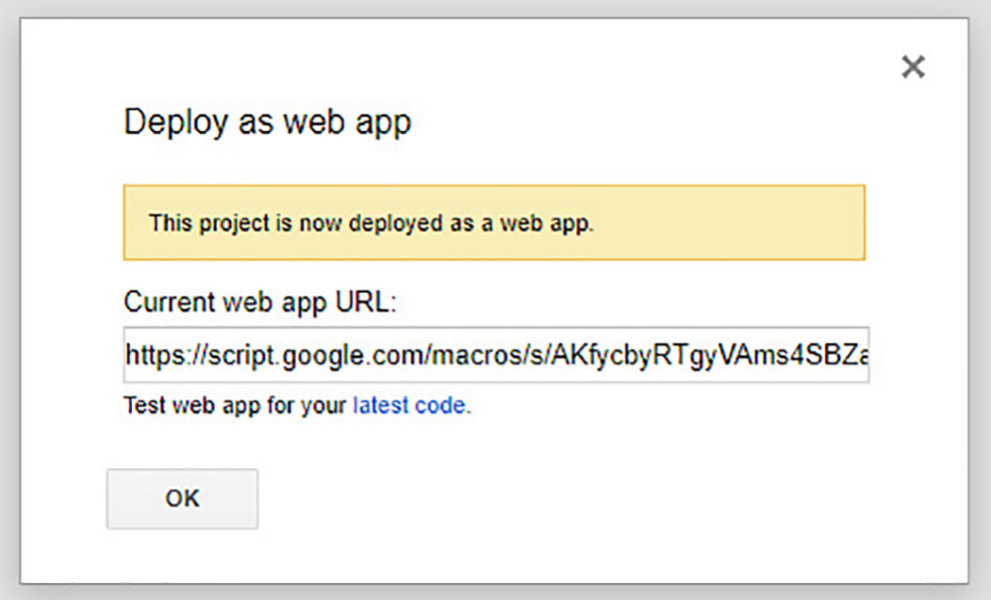
The URL once you deploy and review your account permissions.

Then you will get a pop up that is like Fig. 58**.** Copy the URL that is provided in the pop up and save on notepad.

Once you save it in a separate location, then you can close the Script editor.

**Fig. 59:**
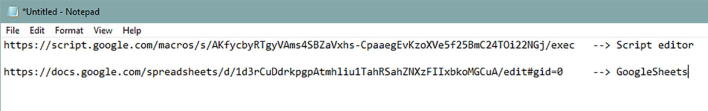
Both URLs used for the next step.

Going back to your GoogleSheets, there is an URL for that GoogleSheets. Copy that and save it in the same location where you save your other URL. But make sure which one is which. These URLs will be used for the next step. For example, this will be what will look in your notepad after saving both URLs. (Fig. 59).

#### 6.3.3 Upload to Hub code on device

**Fig. 60:**
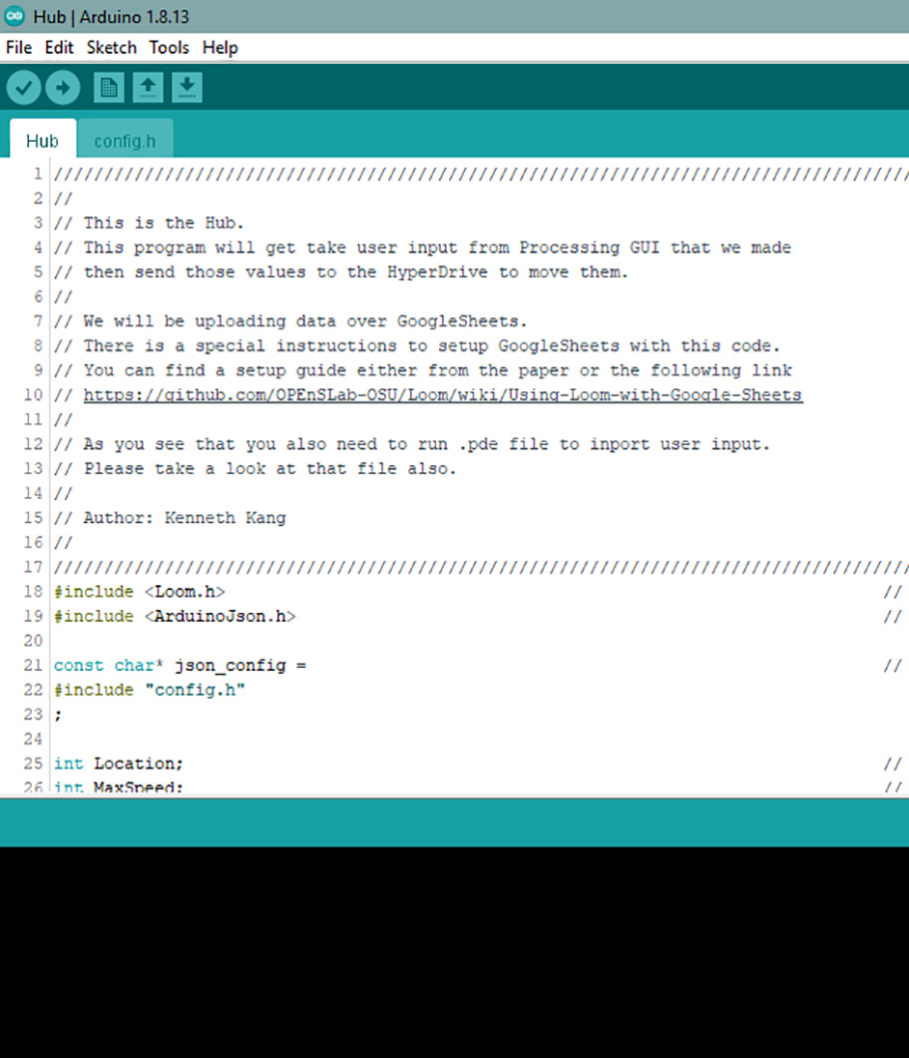
The Hub Code Screen from the Arduino IDE. Make sure to read the description if you are in the correct code.

Within the main project folder, open the Hub folder. In that folder, only open Hub.ino. Once you open it, you will get the screen in Fig. 60.

If you have previously selected the board, it should set as default. If not, then change the board to Loomified Feather M0 like Fig. 47**.**

**Fig. 61:**
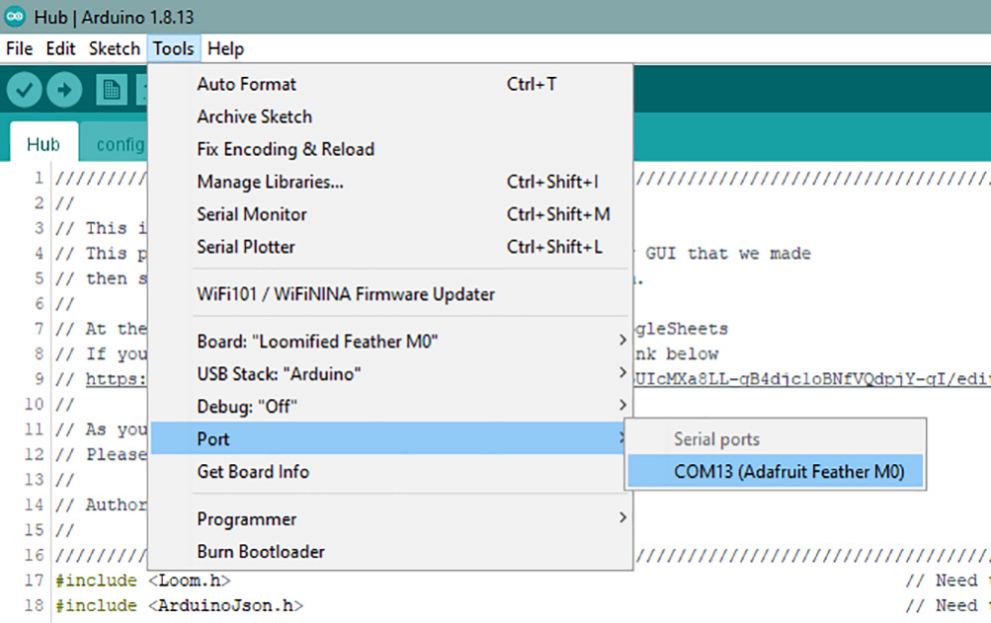
The proper look when you connect the Hub. Note that the port number may be different.

Once that is complete, then connect the Hub to the computer with Micro USB Cable. It should recognize the board. Then go to Tools -> Port and see if there an option to select. If there is no option, try either a different cable or reconnect it. It should be as in Fig. 61. Also, writing down the port number just for the Hub will be useful for later.

**Fig. 62:**
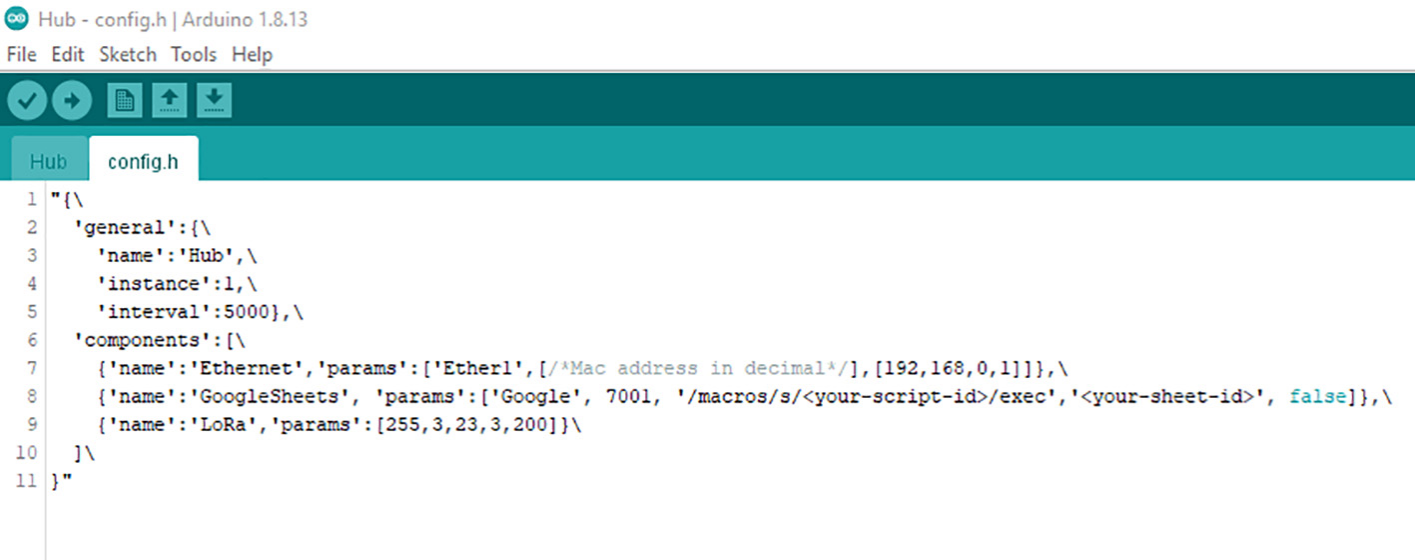
The config.h file for the Hub.ino file.

Once the computer recognizes the board, go to config.h file (Fig. 62).

**Fig. 63:**
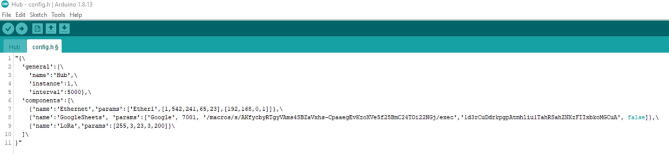
The updated config.h file after entering Mac Address, Script ID, and Sheet ID.

You can see that there are some parts that are commented or in <> (e.g., Fig. 62, line 7). For the Mac address, enter them in decimal. For example, it will be formatted as [1,542,241,65,23] (Fig. 63, line 7). Note that this Mac address is a fake one.

For the < your-script-id>, it will be replaced with the URL from the Script editor. In this example, it will be

AKfycbyRTgyVAms4SBZaVxhs-CpaaegEvKzoXVe5f25BmC24TOi22NGj

Last, <your-sheet-id > will be from the second URL, which it is from the Sheet. In this example, it will be

1d3rCuDdrkpgpAtmhliu1TahRSahZNXzFIIxbkoMGCuA (Fig. 63, line 8).

**Fig. 64:**
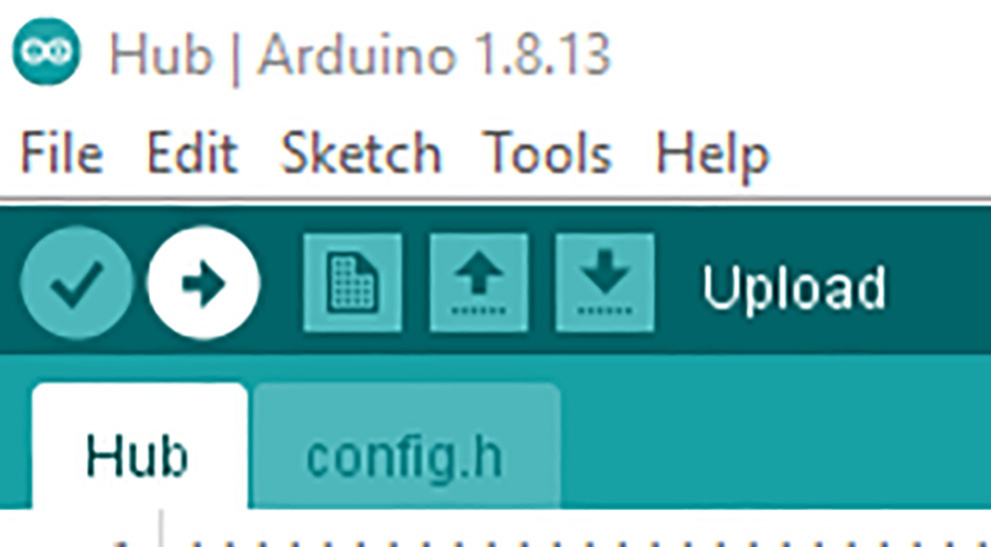
Upload button on the Arduino IDE.

Once you make the changes in the config.h file, save it and click upload using the arrow pointing to the right (Fig. 64).

**Fig. 65:**
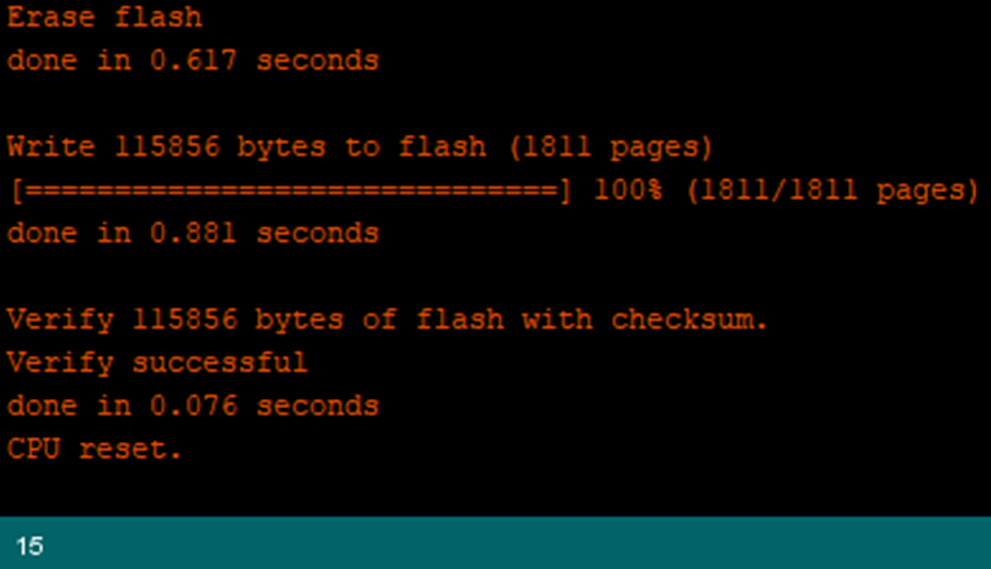
The console log stating that the upload was successful.

Wait until it gets the message shown in Fig. 65. Once you get this message as “CPU Reset”, then it uploaded properly and is ready to use.

### 6.4 HyperRail

**Fig. 66:**
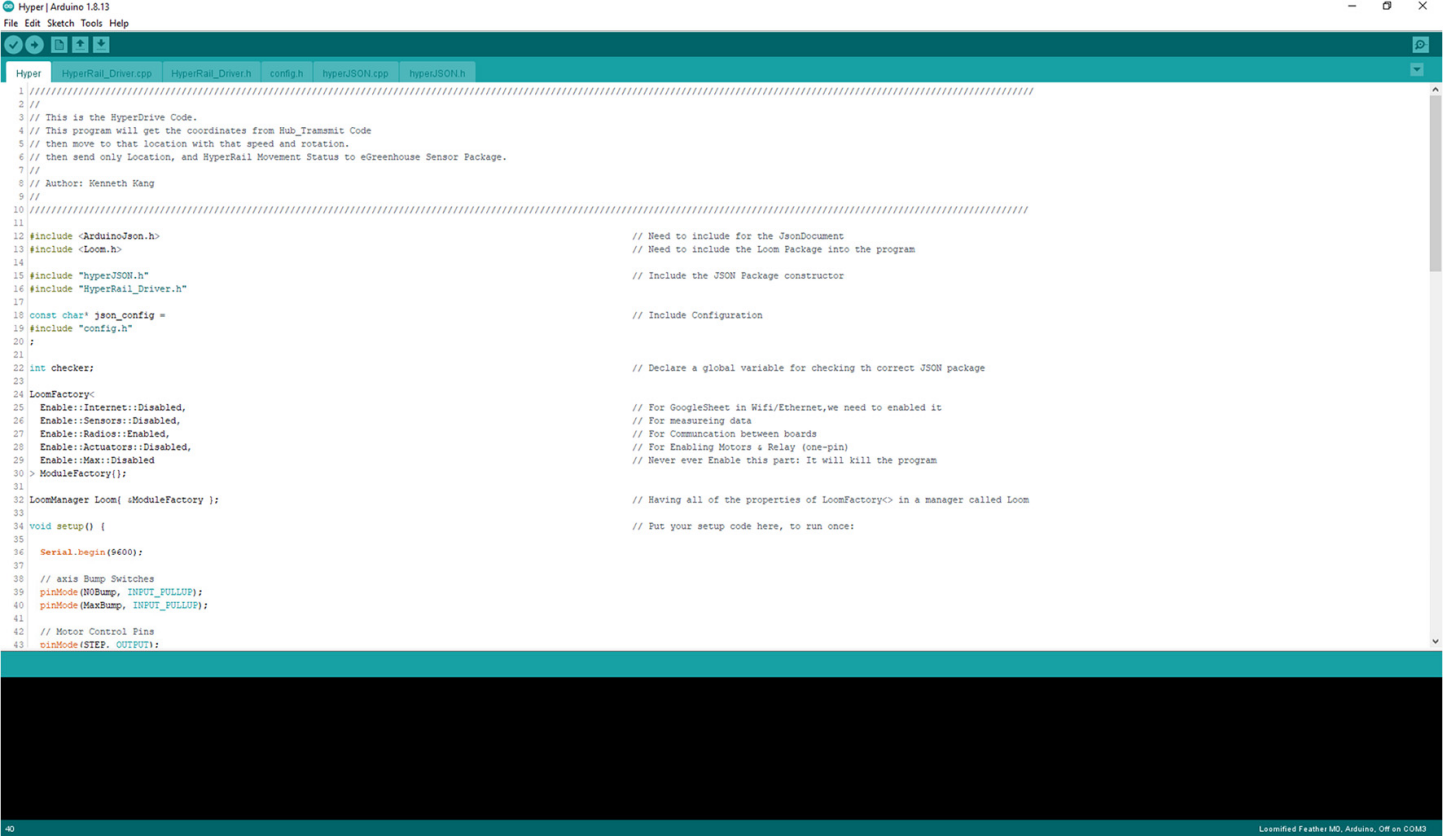
The Hyper Code Screen from the Arduino IDE.

We note that additional description regarding the HyperRail can be found in Lopez et al. [1]. Within the main project folder, open the Hyper folder. In that folder, only open Hyper.ino. Once you open it, you will get the screen shown in Fig. 66.

If you have previously selected the board, it should set as default. If not, then change the board to Loomified Feather M0.

**Fig. 67:**
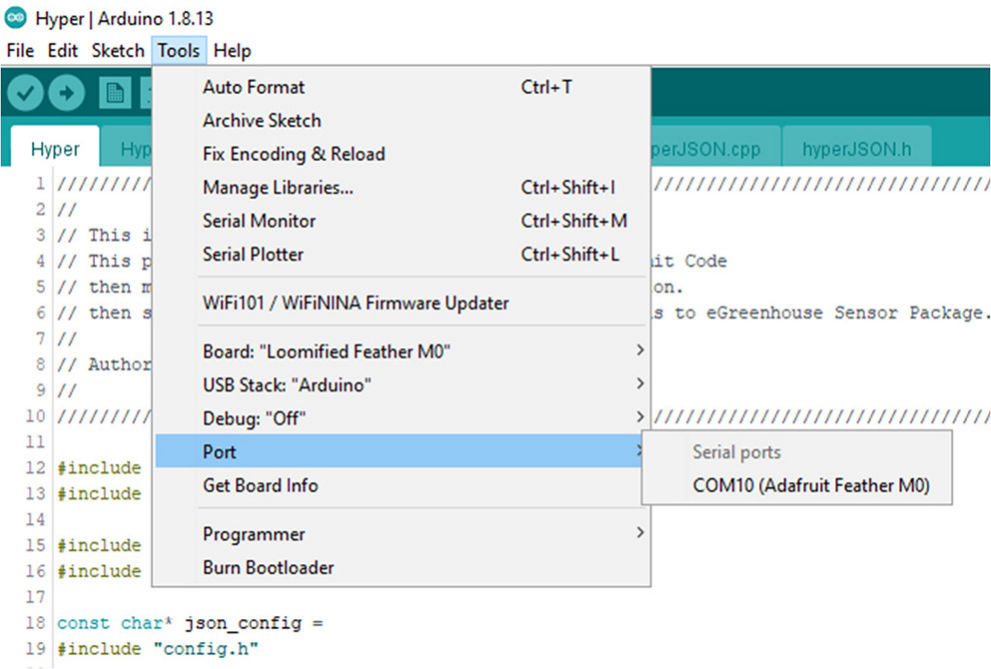
The proper look when you connect the Hyper. Note that the port number may be different.

Once that is complete, then connect the Hyper to the computer with Micro USB Cable. It should recognize the board. Then go to Tools -> Port and see if there is an option to select (Fig. 67). If there is no option, try either a different cable or reconnect it.

**Fig. 68:**
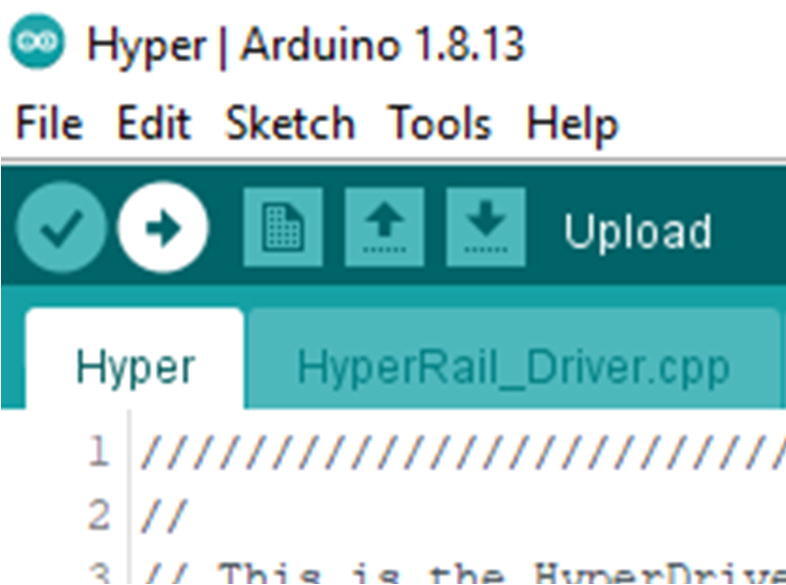
Upload button on the Arduino IDE.

Once the computer recognizes the board, then click upload using the arrow pointing to the right (Fig. 68).

**Fig. 69:**
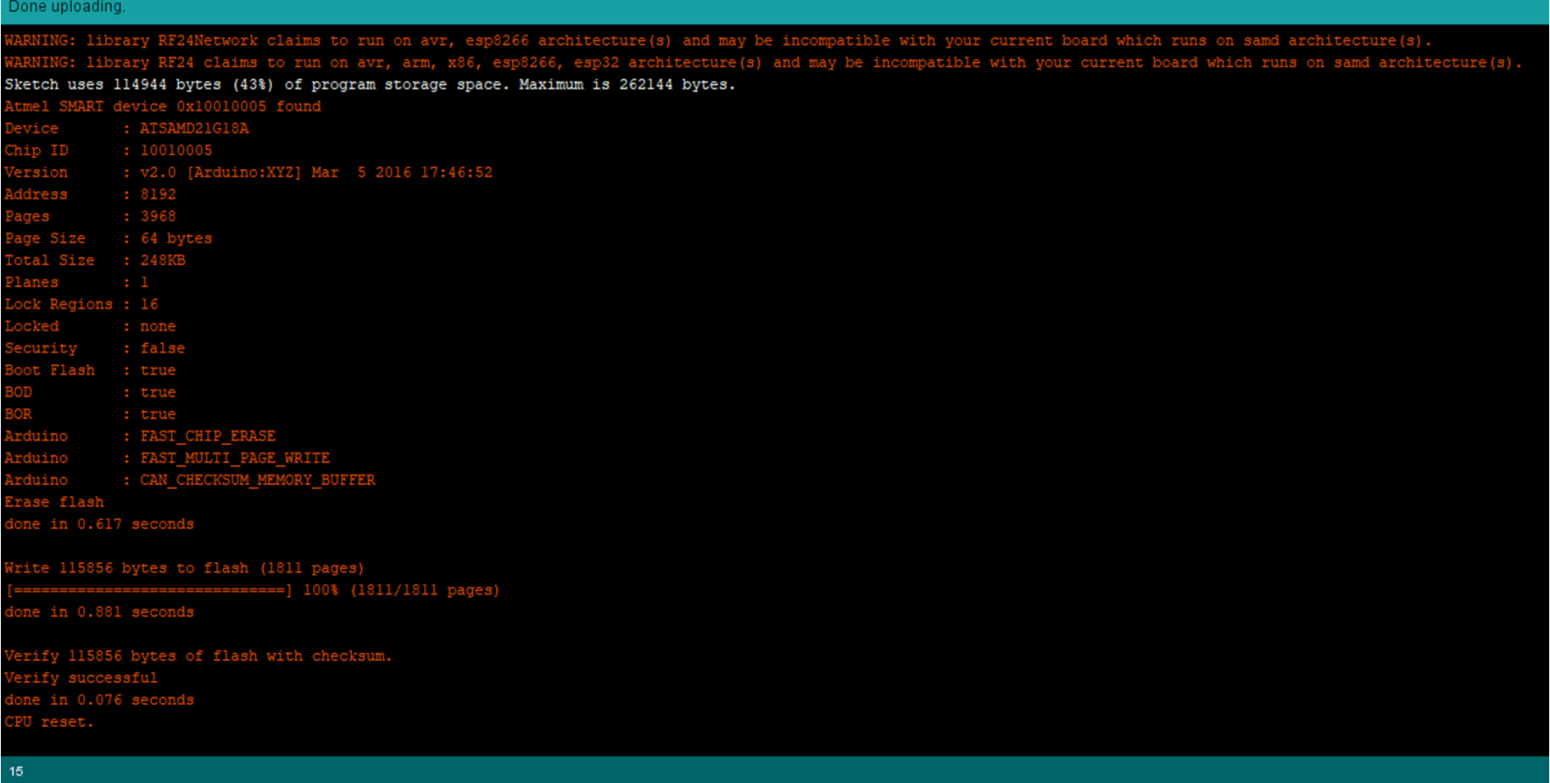
The console log stating that the upload was successful.

Wait until it gets the message shown in Fig. 69. Once you get this message as “CPU Reset”, then it has uploaded properly and is ready to use.

### 6.5 Loading firmware on microcontroller

**Fig. 70:**
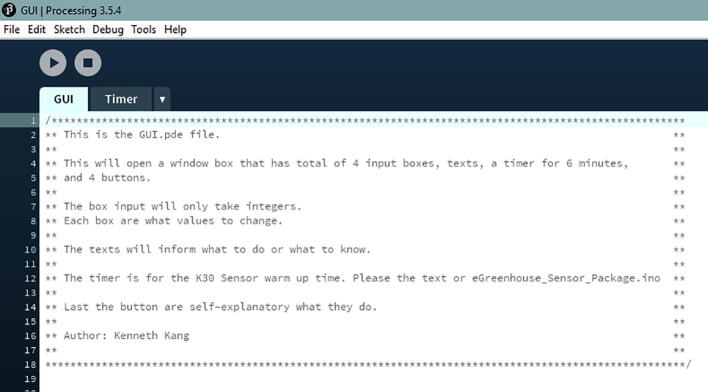
Processing Application for the GUI.

In the design file, open the GUI folder. Inside that folder, only open the GUI.pde file (Fig. 70).

**Fig. 71:**
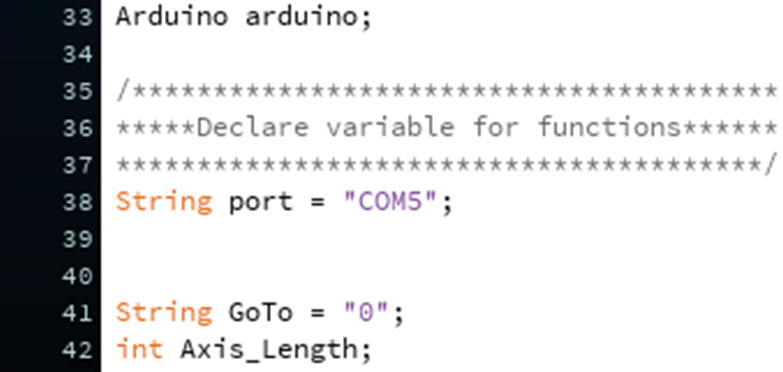
The location where the port number can be changed.

In the code, change the port number for the Hub Board where it is written “String port = “COM5””. You just need to change the number (Fig. 71, line 38). We note that Mac ports show up as USBmodem#### instead of COM# (Windows).

**Fig. 72:**
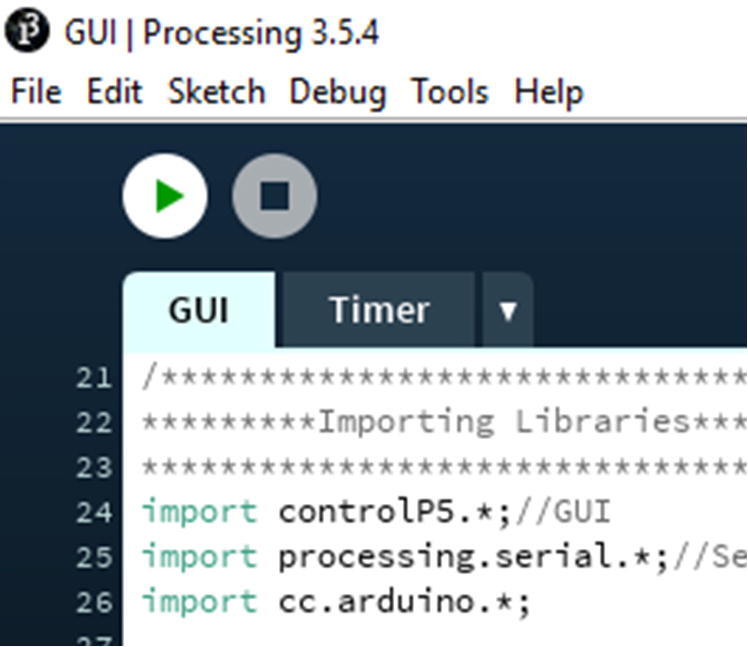
The location to run the Processing Application.

Once that is set, connect the Hub board to the computer and click the green arrow button on the top left (Fig. 72).

**Fig. 73:**
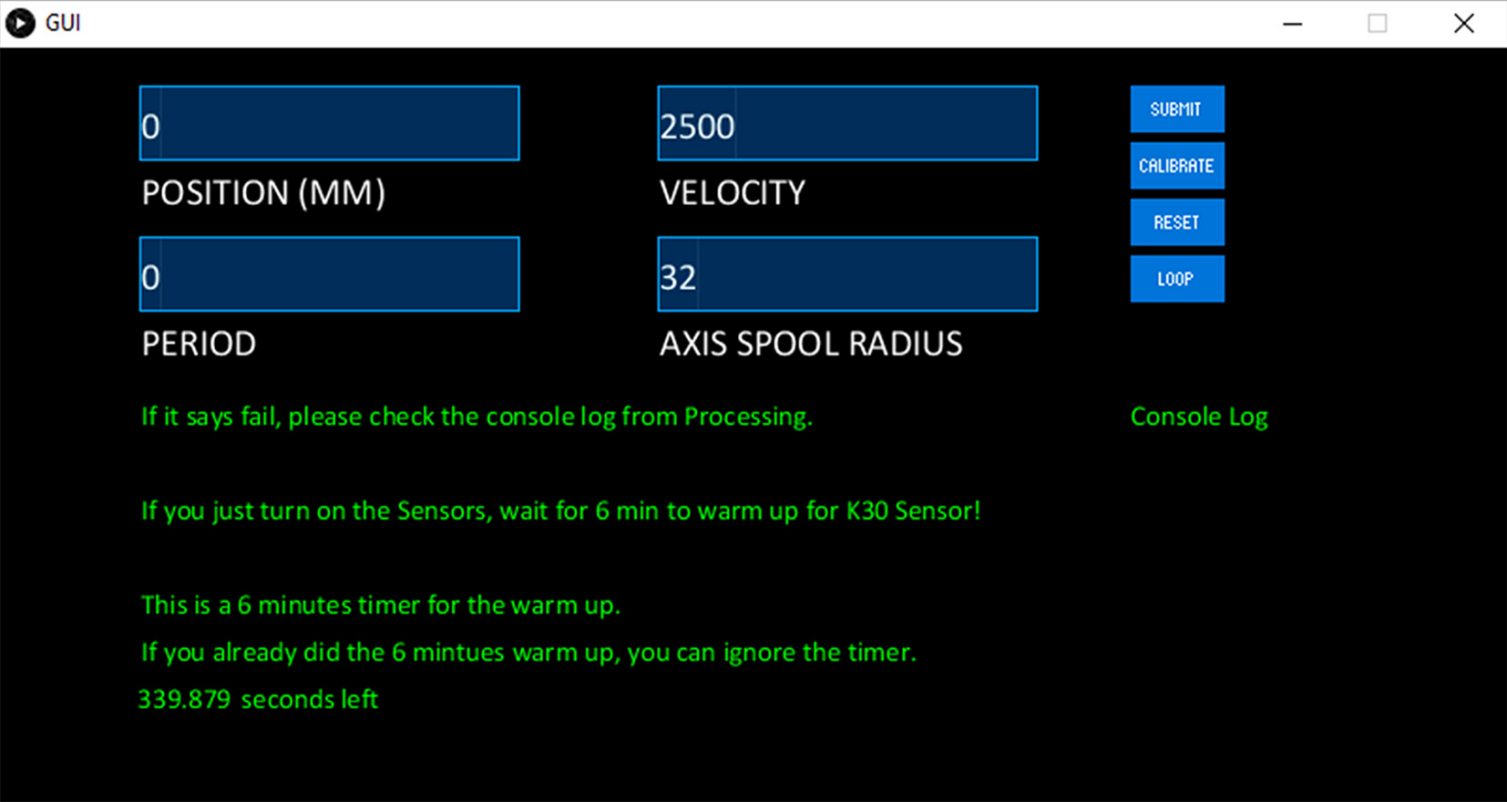
The Graphical User Interface (GUI).

Then you will get this interface as shown Fig. 73.

In the application, you can move the position of the HyperRail, the velocity, the radius (not relevant in the eGreenhouse application), and the duration or number of loops. All the text inputs only take integers. As shown in the GUI, there is a timer for the CO_2_ sensor warm up time. This timer is included because the CO_2_ sensor needs at least 6 min warmup time to get accurate measurements. If the eGreenhouse sensor package was running for more than 6 min, this time can be ignored. On the bottom right, you can see the console log. If the user selects one of the four buttons, it will print out if it transmits to the HyperRail board or not. If not, the failed transmission will be displayed. Once you have successful transmission, you are set to control the HyperRail and measure the eGreenhouse sensor package if both boards are on.

## 7 Validation and characterization

**Fig. 74:**
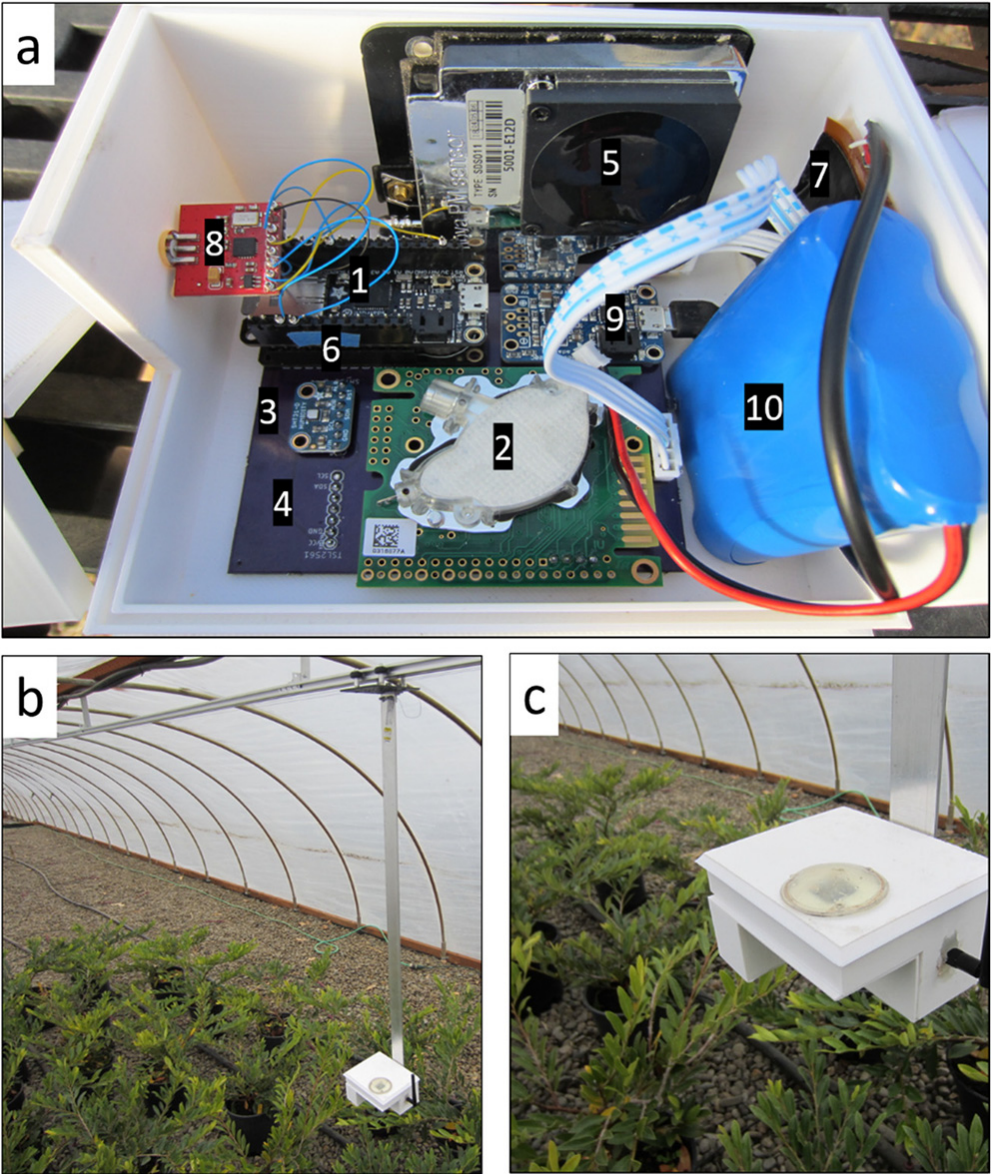
Experimental setting. (a) eGreenhouse hardware – 1. Adafruit Feather Adalogger, 2. CO_2_ sensor Senseair K30, 3. Temperature and relative humidity sensor Adafruit SHT31-D, 4. Light sensor Adafruit TSL2561, 5. Particulate matter sensor Nova PM SDS011 (not operational in this experiment), 6. RTC Adafruit DS3231 Featherwing, 7. Wireless charging Adafruit Universal Qi, 8. Transmission Nordic Semiconductor 2.4 GHz Nrf, 9. Power management Adafruit PowerBoost 1000C, 10. Battery Adafruit 6600 mAh lithium ion battery pack. (b) and (c) are examples of the final configuration mounted on the HyperRail within the greenhouse.

System validation was performed in a full-scale deployment of the eGreenhouse on a 25-meter HyperRail at the North Willamette Research and Extension Center, OR, USA (Fig. 74). The project was conducted in a 30 m (L) × 3 m (W) × 2. 5 m (H) naturally lit, steel framed hoop house (also known as a cold frame). The house was covered with polyethylene plastic, which was rated for 90% light transmissivity. The house was naturally ventilated by raising the plastic on the sidewalls 1 m on both sides of the 30 m length. Due to this specific site requirements, in this eGreenhouse setup we used a nRF radio instead of the LoRa radio with all other key sensors being the same (K30 for CO_2_ and SHT31-D for temperature and relative humidity). Data was sent via nRF across three nodes spanning over 25 m (0, 12.5, and 25 m). The HyperDrive hub [1] drove the sensor package along the rail and sent requests for specified sensors at any spatial interval. Upon receipt of these data, the hub sent the data bundles to a third node at the extension office (~100 m from the greenhouse) with ethernet connectivity. The data was uploaded to Google Sheets, in addition to the onboard SD card logging. Data from the eGreenhouse was compared to data from a nearby meteorological station located ~ 200 m from the greenhouse.

**Fig. 75:**
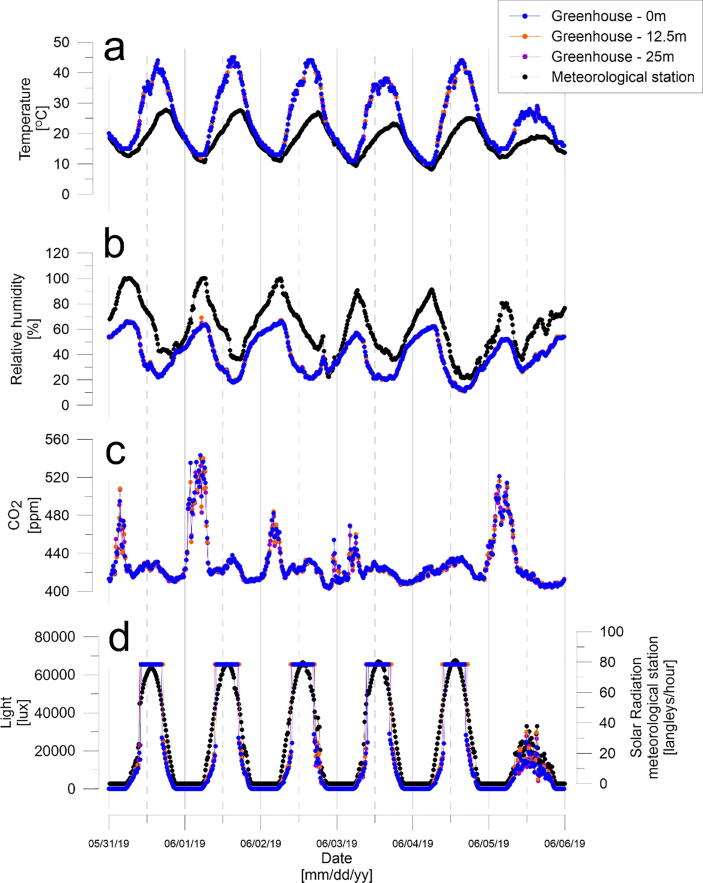
Time series results from six days in the greenhouse and from the meteorological station situated 200 m from the greenhouse.

Data from six consecutive days are presented in Fig. 75. Temperature and relative humidity measured by the eGreenhouse followed the expected trend of higher temperature and lower relative humidity during daytime compared to nighttime (Fig. 75**-a** and **b**). Within the greenhouse, temperatures were higher by ~ 20 °C than the outside temperature during daytime due to the greenhouse effect. No significant spatial differences were found between the three nodes of 0, 12, and 25 m. During nighttime, temperature and relative humidity within the greenhouse were the same as atmospheric values.

CO_2_ values ranged between ~ 400 (atmospheric concentration) and ~ 550 ppm, with higher values during nighttime (Fig. 75**-c**). This is expected because throughout the night the plants respire (CO_2_ is emitted to the greenhouse atmosphere) and photosynthesis, the process by which plants use sunlight and CO_2_ to synthesize food, is suppressed.

**Fig. 76:**
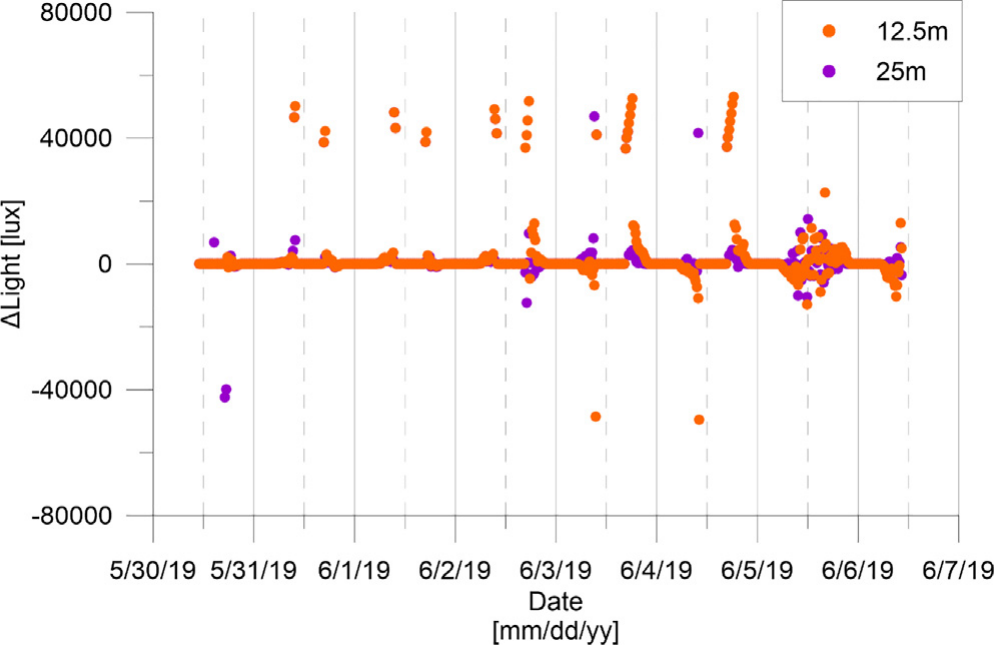
Lux differences between measurements at 12.5 m (orange) or 25 m (purple) and 0 m.

The light values within the greenhouse followed the daily solar cycle (Fig. 75**-d**). Lux values increased with sunrise until reaching sensor saturation each day around noontime and then decreasing after sunset to 0 lux. Light values showed higher spatial distribution than other tested parameters. This is demonstrated by the differences of lux values within the greenhouse between 0, 12.5, and 25 m (Fig. 76). This is most likely due to changes in local shading at each node or as a result of the dynamic cloud movements. The only exception to the daily trend was in the last day (06/05/2019) where light values fluctuated throughout the day without reaching saturation. This day was the cloudiest day of the week, causing lower radiation as measured also by the temperature sensor (Fig. 75**-a**). We note that additional settings and versions of the eGreenhouse are described in the OPEnS lab website (http://www.open-sensing.org/) and GitHub page (https://github.com/OPEnSLab-OSU/eGreenhouse).

## Declaration of Competing Interest

The authors declare that they have no known competing financial interests or personal relationships that could have appeared to influence the work reported in this paper.
